# Anti-adhesive, anti-biofilm and fungicidal action of newly synthesized gemini quaternary ammonium salts

**DOI:** 10.1038/s41598-024-64859-y

**Published:** 2024-06-19

**Authors:** Edyta Mazurkiewicz, Łukasz Lamch, Kazimiera A. Wilk, Ewa Obłąk

**Affiliations:** 1 Department of Physico-Chemistry of Microorganisms, Faculty of Biological Sciences, University of Wrocław, Przybyszewskiego 63/77, 51-148 Wrocław, Poland; 2 Department of Engineering and Technology of Chemical Processes, Faculty of Chemistry, Wrocław University of Science and Technology, Wybrzeże Wyspiańskiego 27, 50-370 Wrocław, Poland

**Keywords:** Biological techniques, Microbiology, Medical research

## Abstract

Newly synthesized gemini quaternary ammonium salts (QAS) with different counterions (bromide, hydrogen chloride, methylcarbonate, acetate, lactate), chain lengths (C12, C14, C16) and methylene linker (3xCH_2_) were tested. Dihydrochlorides and dibromides with 12 carbon atoms in hydrophobic chains were characterized by the highest biological activity against planktonic forms of yeast and yeast-like fungi. The tested gemini surfactants also inhibited the production of filaments by *C. albicans*. Moreover, they reduced the adhesion of *C. albicans* cells to the surfaces of stainless steel, silicone and glass, and slightly to polystyrene. In particular, the gemini compounds with 16-carbon alkyl chains were most effective against biofilms. It was also found that the tested surfactants were not cytotoxic to yeast cells. Moreover, dimethylcarbonate (2xC_12_MeCO_3_G_3_) did not cause hemolysis of sheep erythrocytes. Dihydrochlorides, dilactate and diacetate showed no mutagenic potential.

## Introduction

Gemini (dimeric) surfactants have provided a lot of advantages of their unique combination of physical and chemical properties and being used in ordinary household objects to multifarious industrial processes. They were shown to reveal enhanced thermal and surface properties in comparison to their monomeric counterparts. Performance evaluation of such custom-designed products showed superiority in terms of surface tension, foaming, emulsification ability, and lime soap dispersibility. The research on interfacial and biological properties of multifunctional cationic surfactants is very important from a practical point of view mainly in petroleum, chemical and pharmaceutical industries. In addition, their desirable rheological characterization and interfacial tension are two of the most applicable screening techniques for the evaluation and selection of chemicals for enhanced oil recovery ^1–4^. A wide range of applications is connected with their adsorption and aggregation properties, high viscosity, detergency, solubilization ability, improvement of wetting and profound antimicrobial activity against bacteria, yeasts and yeast—like fungi. Among them, gemini (dimeric) derivatives, consisting of two hydrophobic chains and two quaternary ammonium headgroups linked by a rigid or flexible spacer, seem to be the most interesting in view of their tunable molecular geometry and aggregate morphology. The current literature data makes one to estimate the effect of structural variation on the aggregation behavior of gemini surfactants for nanoscience and biological applications like antimicrobial, anti-fungal agent, better gene and drug delivery agent with low cytotoxicity and biodegradability, which makes them more advantageous for a number of technological processes and hence reduces by rationally-designed architecture the impact of these gemini surfactants on the environment ^3,5–7^.

The properties of gemini (or dimeric) surfactants are much better than those of conventional ammonium salts. A broad review of interfacial properies of quaternary gemini surfactants has been done by Zana ^8,9^. Their surface activity was found to be 1–2 orders of magnitude higher than the surface activity of the corresponding monomeric surfactants. They show excellent surface-active properties, revealing a strong tendency to adsorb at air–water interfaces (form denser layers), provide satisfactory efficiency in lowering the surface tension, and form micelles in water at a relatively low concentrations—they exhibit a much lower critical micelle concentration (cmc) and good water solubility, unusual micelle structures and aggregation behavior ^10,11^. Dimeric surfactants also have a higher antimicrobial activity and a broader spectrum of action compared to monomeric surfactants ^12,13^. Their effectiveness depends on the length of the hydrophobic chain and the spacer. Generally, it can be concluded, however, that the dominant role in outstanding antimicrobial property for varieties of bacteria was displayed by the hydrophobic chains length ^14^.

The biological properties of gemini surfactants can be successfully applied in medicine (as disinfectants, drugs, and DNA carriers), industry, environmental protection and agriculture (as preservatives, biocides, herbicides and fungicides). Such compounds have the ability to coat various surfaces, preventing the adhesion of microorganisms and destroying biofilms created by pathogens. However, some biofilms produced by pathogens are resistant to commonly used disinfectants ^15^. The chemical and biological properties of these surfactants depend on their chemical structure (e.g. the length of hydrophobic chains and linkers and counterions) ^6,14,16^. QAS, due to amphiphilic nature, interact with the biological membrane of microorganisms, leading to disruption of its permeability and, consequently, to cell death ^15^.

Moreover, these surfactants have the ability to form stable complexes with DNA (lipoplexes). Therefore, they can be used in medicine as non-viral gene or drug carriers ^17–23^. These compounds appear to be efficacious agents in mediating transfection. Such carriers can carry DNA fragments of various sizes and, unlike viral vectors, don’t cause an immune response or insertional mutagenesis ^22–24^.

One of the most important surfactants in view of nowadays requirements as so-called soft structures, denoted in the literature as chemo-degradable, destructible, hydrolysable and acid (alkali) sensitive ^25–29^, whose hydrophobic parts are functionalized with structural labile/cleavable acetal, amide, amine, ester, disulphide, ether or thioether moieties. Chemically and/or enzymatically invoked cleavage of the mentioned grouping may cause the change of surface activity as a result of the separation of the polar part and the hydrophobic tail ^29^. In recent years, environmental concerns coupled with increased consumer awareness have guided substantial growth of environmentally benign surfactant molecules ^30^ and the soft-type products may fulfill these requirements. Moreover, these products, depending on its type, number, and positions of the inserted functional groups, may possess new amenable physicochemical and biological functionality, as well as improved performance ^31–33^. The most commercially viable example of soft surfactants comprises the family of cationic amido/ester-containing representatives with the labile bond inserted between the hydrocarbon tail and the quaternary ammonium head group ^34,35^. The gemini-type cationic surfactants with different hydrophobic alkyl chain lengths that are the main objectives in the present study comprise the labile amide-type grouping.

The main aim of the present study was to design, synthesize and carefully analyze, in terms of both physicochemical and biological activity, novel class of gemini cationic surfactants, comprising: (1) sufficient stability and moderate ability to undergo biodegradation and/or chemo-degradation; (2) gemini-type architecture with spacer between linking groups combining hydrophilic and hydrophobic counterparts; (3) nitrogen atom bearing positive electric charge as part of hydrophilic group as well as (4) profound anti-adhesive, anti-biofilm and fungicidal action against various strains. Taking into account the aforementioned issues we have designed novel class of quaternary ammonium-type cationic surfactants, derivatives of N1-(3-(3-(dimethylamino)propylamino) propyl)-N3,N3-dimethylpropane-1,3-diamine—tetraamine with two secondary and two tertiary amine groups, separated by three trimethylene motifs—comprising two alkyl chains, attached to the hydrophilic groups and trimethylene spacer by tetriary amide motifs. It should be emphasized, that use of tertiary amide groups comprise a kind of compromise between biodegradability and stability in aqueous solution—in contrast to easily undergoing hydrolysis ester and secondary amide motifs such linking moieties could be sufficiently long dissolved without significant degradation.

In this study, we investigated the biological activity of newly designed and synthesized gemini QAS with different chemical structures (different lengths of hydrophobic chains, different counterions and a linker containing three methylene groups) against yeast and yeast-like fungi, both in the planktonic and biofilm form.

## Results

### Design and synthesis of gemini quaternary ammonium type surfactants

**Figure 1:**
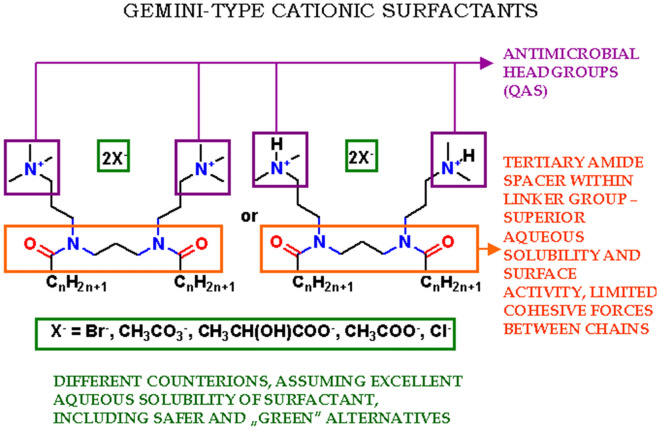
The general idea of our gemini quaternary-type cationic surfactants: derivatives with dodecyl (n = 11), tetradecyl (n = 13) and hexadecyl (n = 15) alkyl chain.

Our newly devised compounds were designed in order to meet requirements of novel surfactants, especially comprising balanced stability—biodegradability (use of tertiary amide linker), novel counterions (beside the most common dibromides and dihydrochlorides novel milder dimethylcarbonates, dilactates and diacetates have been designed and synthesized). Such considerations were aimed to assume superior aqueous solubility of gemini-type surfactant by moderation of hydrogen bonding within linker moiety (use of tertiary amide instead of typically occurring secondary one) as well as limitation of cohesive forces between two alkyl chains. For the design of our surfactants see Fig. 1.

**Figure 2:**
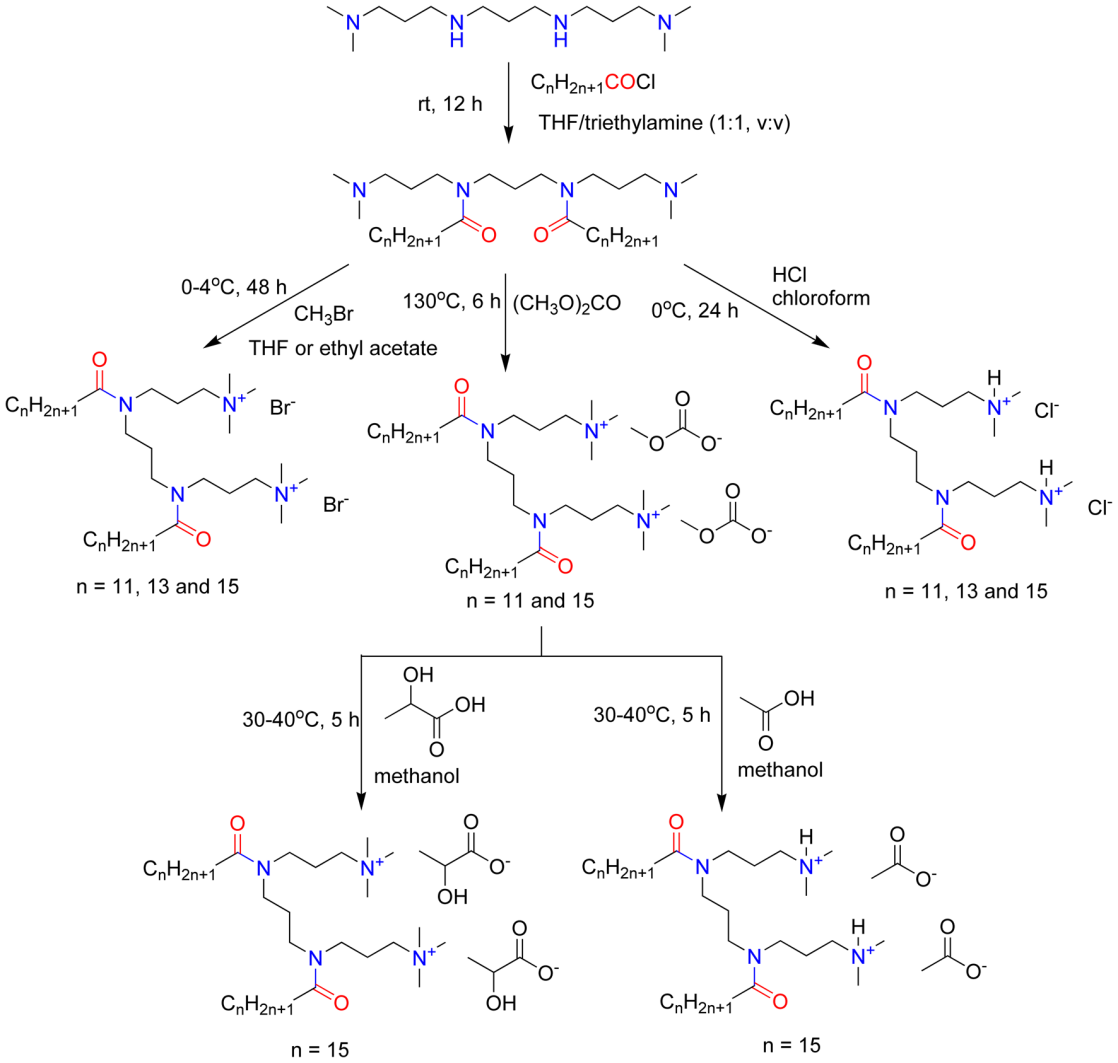
The synthetic routes of our gemini quaternary-type cationic surfactants.

Generally, synthesis of gemini type surfactants constitute a multistep route, comprising main steps as formation of hydrophobic or hydrophilic fragments precursors, followed by their coupling and quaternization, for cationic or amphotheric derivatives. For our gemini-type surfactants we have chosen synthetic route comprising formation of hydrophobic “skeleton” of the surfactant molecule, followed by its appropriate hydrophilization by reaction with excess of hydrogen chloride (for dihydrochlorides), bromomethane (for dibromides) or dimethyl carbonate (for dimethylcarbonates). Appropriate dimethylcarbonates were converted into dilactates and diacetates by counterion exchange in anhydrous environment with lactic or acetic acid, respectively. See Fig. 2 for the general synthetic route of our surfactants.

The first step, i.e. synthesis of diamidediamine hydrophobic derivative, was performed in tetrahydrofuran—trimethylamine mixture with use of N^1^-(3-(3-(dimethylamino)propylamino)propyl)-N^3^,N^3^-dimethylpropane-1,3-diamine and appropriate alkanoyl chloride as substrates in molar ratio 1:2, respertively. The side product (trimethylamine hydrochloride) was removed by filtration, while solvents evaporation yielded stoichiometrically appropriate intermediate. The letter compounds were quaternized or coupled with hydrogen chloride in appropriate solution (aprotic solvent for reaction with bromomethane, polar environment for reaction with hydrogen chloride or quaternizning agent excess for dimethyl carbonate), followed be careful, repeated crystallization (total yield of purified products: 65–90%). It should be noted that our surfactants were obtained as hygroscopic solids, therefore careful drying and storage in desiccator were needed. Moreover counterion exchange for dilactates and diacetates needed to be performed in completely dry methanol under atmosphere of inert gas, followed by careful crystallization and drying of the devised products. The chemical structures of the obtained surfactants were confirmed by ^1^H NMR, ^13^C NMR and ESI–MS analyses, while their purities by elemental analyses—see Table 1 and Fig. 1S–14S in ESI. All of the compounds were characterized by Krafft points—see Table 1—below room temperature, while all compounds with dodecyl and tetradecyl alkyl chain had this parameter below 0 °C. Such characteristics indicate excellent aqueous solubility—typical cationic surfactants possess Krafft points above 25 °C for hexadecyl or longer chains ^2^. Moreover, due to strict relationship between Krafft points and CMC values, surfactants with Krafft points below room temperature can be studied in both micellar (concentrated) and unimolecular (diluted) states.

**Table 1:** Properties and spectroscopic data of the studied gemini quaternary ammonium-type surfactants.

Chemical structure (Abbreviation)	Molecular weight [g/mol]	^1^H NMR, CDCl_3_	^13^C NMR, CDCl_3_	ESI–MS (M^+^)	T_K_ [°C]	Elemental analyses (calculated)			CMC [mol/dm^3^]
		δ [ppm]	δ [ppm]			%C	%H	%N	
(2xC_n_BrG_3_; n = 12, 14, 16)	789.89	0.83–0.89 [t, 2x3H, –COCH_2_CH_2_(CH_2_)_k_**CH**_**3**_]; 1.20–1.35 [m, 2x2kH, –COCH_2_CH_2_**(CH**_**2**_**)**_**k**_CH_3_]; 1.52–1.62 [m, 2x2H, –COCH_2_**CH**_**2**_(CH_2_)_k_CH_3_]; 1.81–2.06 [m, 2H, 2x2H –N^+^CH_2_**CH**_**2**_CH_2_N–, –CH_2_**CH**_**2**_CH_2_N–]; 2.21–2.33 [t, 2x2H, –CO**CH**_**2**_CH_2_(CH_2_)_k_CH_3_]; 3.22–3.44 [m, 2x4H, 2x11H; –**CH**_**2**_N**CH**_**2**_–,—**CH**_**2**_N^+^(**CH**_**3**_)_3_]	14 [–(CH_2_)_k_**C**H_3_]; 20–35 [–**C**H_2_–]; 44–47 [–N**C**H_2_–] 55 [–N^+^(**C**H_3_)_3_]; 65 [–**C**H_2_N^+^]; 170 [> **C** = O]	710.0	< 0	59.25 (59.30)	10.45 (10.49)	7.12 (7.09)	6.4*10^–5^
	855.01			755.1	< 0	60.62 (60.40)	10.41 (10.63)	6.54 (6.55)	5.4*10^–6^
	911.12			831.2	~ 5	61.88 (61.95)	10.83 (10.86)	6.12 (6.15)	4.6*10^–7^
(2xC_n_HClG_3_; n = 12, 14, 16)	681.95	0.83–0.89 [t, 2x3H, –COCH_2_CH_2_(CH_2_)_8_**CH**_**3**_]; 1.20–1.35 [m, 2x2kH, –COCH_2_CH_2_**(CH**_**2**_**)**_**k**_CH_3_]; 1.52–1.62 [m, 2x2H, –COCH_2_**CH**_**2**_(CH_2_)_k_CH_3_]; 1.81–2.06 [m, 2H, 2x2H –N^+^CH_2_**CH**_**2**_CH_2_N–, –NCH_2_**CH**_**2**_CH_2_N–]; 2.21–2.35 [t, 2x2H, –CO**CH**_**2**_CH_2_(CH_2_)_k_CH_3_]; 3.09–3.27 [m, 2x4H, 2x8H; –**CH**_**2**_N**CH**_**2**_–,—**CH**_**2**_N^+^(**CH**_**3**_)_2_]	14 [–(CH_2_)_k_**C**H_3_]; 20–35 [–**C**H_2_–]; 44–57 [–N**C**H_2_–, –N(**C**H_3_)_2_]; 170 [> **C** = O]	646.5	< 0	65.29 (65.16)	11.39 (11.55)	8.28 (8.22)	2.2*10^–4^
	738.05			702.6	< 0	66.61 (66.72)	11.89 (11.77)	7.65 (7.59)	1.9*10^–5^
	794.16			758.7	~ 15	68.07 (68.05)	11.92 (11.95)	7.05 (7.06)	1.6*10^–6^
(2xC_n_MeCO_3_G_3_; n = 12, 16)	789.18	0.85–0.90 [t, 2x3H, –COCH_2_CH_2_(CH_2_)_k_**CH**_**3**_]; 1.22–1.34 [m, 2x2kH, –COCH_2_CH_2_**(CH**_**2**_**)**_**k**_CH_3_]; 1.55–1.60 [m, 2x2H, –COCH_2_**CH**_**2**_(CH_2_)_k_CH_3_]; 1.80–2.03 [m, 2H, 2x2H –N^+^CH_2_**CH**_**2**_CH_2_N–, –NCH_2_**CH**_**2**_CH_2_N–]; 2.25–2.34 [t, 2x2H, –CO**CH**_**2**_CH_2_(CH_2_)_k_CH_3_]; 3.20–3.45 [m, 2x4H, 2x11H; –**CH**_**2**_N**CH**_**2**_–,—**CH**_**2**_N^+^(**CH**_**3**_)_3_]; 3.64–3.67 [m, 2x3H;—C**H**_**3**_CO_3_^–^]	14 [–(CH_2_)_k_**C**H_3_]; 20–35 [–**C**H_2_–]; 44–47 [–N**C**H_2_–] 55 [–N^+^(**C**H_3_)_3_, –O**C**H_3_]; 65 [–**C**H_2_N^+^]; 160–172 [> **C** = O]	714.1	< 0	65.59 (65.44)	11.12 (11.26)	7.19 (7.10)	1.8*10^–4^
	901.39			826.4	~ 5	68.11 (67.95)	11.51 (11.65)	6.26 (6.22)	1.6*10^–6^
(2xC_n_LaG_3_; n = 16)	929.45	0.84–0.89 [t, 2x3H, –COCH_2_CH_2_(CH_2_)_12_**CH**_**3**_]; 1.20–1.34 [m, 2x16H, –COCH_2_CH_2_**(CH**_**2**_**)**_**12**_CH_3_]; 1.54–1.62 [m, 2x2H, –COCH_2_**CH**_**2**_(CH_2_)_12_CH_3_]; 1.81–2.09 [m, 2H, 2x2H –N^+^CH_2_**CH**_**2**_CH_2_N–, –CH_2_**CH**_**2**_CH_2_N–]; 2.21–2.32 [t, 2x2H, –CO**CH**_**2**_CH_2_(CH_2_)_12_CH_3_]; 3.05–3.30 [m, 2x4H, 2x11H, 2x3H; –**CH**_**2**_N**CH**_**2**_–,—**CH**_**2**_N^+^(**CH**_**3**_)_3,_ C**H**_**3**_CH(OH)COO^–^]; 4.25–4.30 [m, 2x1H; CH_3_C**H**(OH)COO^–^]	14 [–(CH_2_)_k_**C**H_3_]; 20–35 [–**C**H_2_–, > CH**C**H_3_]; 44–47 [–N**C**H_2_–]; 55 [–N^+^(**C**H_3_)_3_]; 65 [–**C**H_2_N^+^]; 78 [> **C**HOH]; 170–178 [> **C** = O]	840.4	< 0	68.74 (68.48)	11.58 (11.74)	6.11 (6.03)	3.7*10^–7^
(2xC_n_AcG_3_; n = 16)	869.40	0.85–0.88 [t, 2x3H, –COCH_2_CH_2_(CH_2_)_12_**CH**_**3**_]; 1.22–1.33 [m, 2x16H, –COCH_2_CH_2_**(CH**_**2**_**)**_**12**_CH_3_]; 1.50–1.60 [m, 2x2H, –COCH_2_**CH**_**2**_(CH_2_)_12_CH_3_]; 1.83–2.12 [m, 2H, 2x2H –N^+^CH_2_**CH**_**2**_CH_2_N–, –CH_2_**CH**_**2**_CH_2_N–]; 2.19–2.31 [t, 2x2H, –CO**CH**_**2**_CH_2_(CH_2_)_12_CH_3_]; 3.11–3.23 [m, 2x4H, 2x11H, 2x3H; –**CH**_**2**_N**CH**_**2**_–,—**CH**_**2**_N^+^(**CH**_**3**_)_3,_ C**H**_**3**_COO^–^]	14 [–(CH_2_)_k_**C**H_3_]; 20–35 [–**C**H_2_–, –CO**C**H_3_]; 44–47 [–N**C**H_2_–]; 55 [–N^+^(**C**H_3_)_3_, –O**C**H_3_]; 65 [–**C**H_2_N^+^]; 170–173 [> **C** = O]	810.4	~ 5	70.59 (70.45)	12.17 (12.08)	6.50 (6.45)	2.2*10^–6^

### Determination of minimum inhibitory concentration and fungicidal concentration of the tested compounds

In order to determine the biological activity of the newly synthesized gemini QAS, first of all, their minimum growth inhibitory concentration and minimum fungicidal concentration against yeast strains and yeast-like fungi were determined (Table 2).

**Table 2:** Minimum inhibitory concentration (MIC) and fungicidal concentration (MFC) of tested gemini QAS against selected microorganisms.

Strain	Minimum inhibitory (MIC) and fungicidal (MFC*) concentration [µM] of gemini QAS with different counterions (bromide—Br, methylcarbonate—MeCO_3_, hydrochloride—HCl, lactate—La, acetate—Ac), different alkyl chain lengths (C12, C14, C16) and a methylene linker G_3_ (3xCH_2_)									
	Dibromides			Dihydrochlorides			Dimethylcarbonates		Dilactates	Diacetates
	2xC_12_BrG_3_	2xC_14_BrG_3_	2xC_16_BrG_3_	2xC_12_HClG_3_	2xC_14_HClG_3_	2xC_16_HClG_3_	2xC_12_MeCO_3_G_3_	2xC_16_MeCO_3_G_3_	2xC_16_LaG_3_	2xC_16_AcG_3_
*Saccharomyces cerevisiae* Σ1278b ATCC 42,800	240 320*	360 640*	> 1280 > 1280*	160 320*	320 640*	> 1280 > 1280*	640 > 1280*	> 1280 > 1280*	> 1280 > 1280*	> 1280 > 1280*
*Saccharomyces cerevisiae* BY4741 ATCC 201,388	20 320*	160 1280*	320 > 1280*	20 640*	160 640*	640 > 1280*	160 1280*	640 1280*	640 > 1280*	> 1280 > 1280*
*Candida albicans* ATCC 10,231	640 640*	640 1280*	> 1280 > 1280*	160 640*	160 640*	> 1280 > 1280*	> 1280 > 1280*	> 1280 > 1280*	> 1280 > 1280*	> 1280 > 1280*
*Candida albicans* 595/20	1280 > 1280*	640 > 1280*	> 1280 > 1280*	640 > 1280*	320 > 1280*	> 1280 > 1280*	> 1280 > 1280*	> 1280 > 1280*	> 1280 > 1280*	> 1280 > 1280*
*Rhodotorula mucilaginosa* ATCC 4056	10 10*	80 320*	> 640 640*	2,5 5*	40 320*	40 640*	320 320*	640 640*	80 640*	> 1280 > 1280*

The best inhibitory (MIC) and fungicidal (MFC) activity against yeast and yeast-like fungi, among the tested gemini QAS, was shown by dihydrochlorides (2xC_12_HClG_3_) and dibromides (2xC_12_BrG_3_). The MIC of dihydrochloride (2xC_12_HClG_3_) against the *R. mucilaginosa* strain was 2.5 μM and that of dibromide (2xC_12_BrG_3_) was 10 μM. However, against *S. cerevisiae* BY4741 their MIC was 20 μM. The fungicidal effect of other surfactants—dibromides (2xC_14_BrG_3_, 2xC_16_BrG_3_), dimethylcarbonates (2xC_12_MeCO_3_G_3_, 2xC_16_MeCO_3_G_3_), dihydrochlorides (2xC_14_HClG_3_, 2xC_16_HClG_3_) and dilactates (2xC_16_LaG_3_)—was weaker. Their MIC and MFC started from 40 μM against *R. mucilaginosa* and from 160 μM against other strains. Diacetate with 16-carbon alkyl chains had the weakest effect. This compound, in the selected concentration range, did not show any inhibitory effect on the tested strains (Table 2).

### Filamentation

Filaments produced by microorganisms such as *C. albicans* are structures that facilitate the adhesion of pathogens to surfaces and the formation of biofilms.

**Figure 3:**
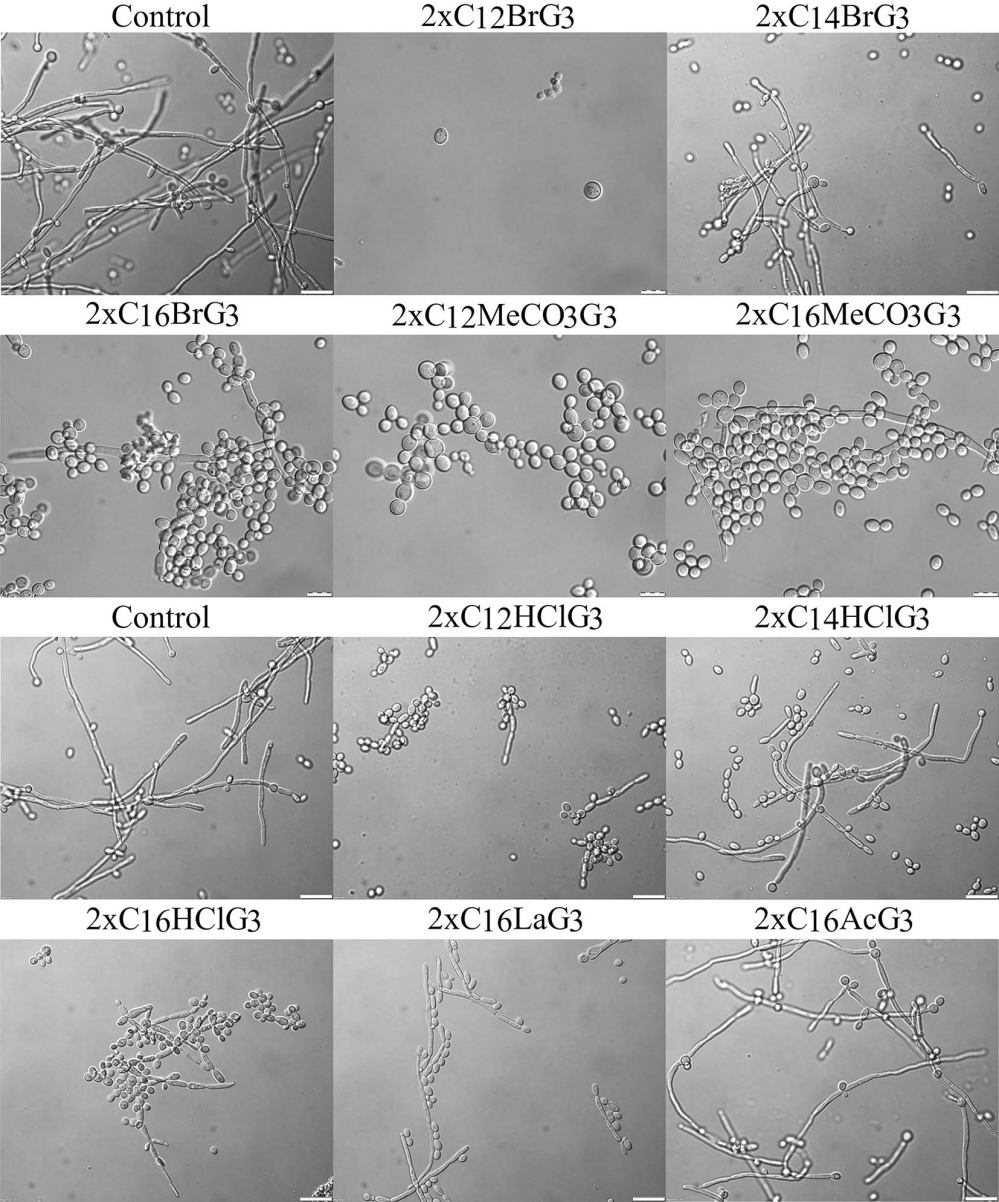
Filamentation of *C. albicans* cells after 24-h incubation, under control conditions (without QAS) and in the presence of the tested surfactants ((2xC_12_BrG_3_, 2xC_14_BrG_3_, 2xC_16_BrG_3_, 2xC_12_MeCO_3_G_3_, 2xC_16_MeCO_3_G_3_, 2xC_12_HClG_3_, 2xC_14_HClG_3_, 2xC_16_HClG_3_, 2xC_16_LaG_3_, 2xC_16_AcG_3_). Scale bar = 20 μm.

The effect of the tested gemini QAS on the production of filaments by *C. albicans* ATCC 10231 was examined after 24 h of incubation (Fig. 3). Dibromides with 12-carbon hydrophobic chains completely inhibits filament formation and cell growth. However, dibromides with 16-carbon alkyl chains largely prevented the formation of filaments. Dimethylcarbonates (2xC_12_MeCO_3_G_3_) almost completely inhibited the production of filaments, and dimethylcarbonates (2xC_16_MeCO_3_G_3_) significantly reduced their number. Dibromides with 14-carbon alkyl chains had little effect on the formation of filaments. In turn, dihydrochlorides with 12-, 14- and 16-carbon alkyl chains significantly inhibit the formation of these structures, with the strongest effect shown by surfactants with 12-carbon hydrophobic chains. Dilactate (2xC_16_LaG_3_) also strongly prevented the formation of filaments, while diacetate (2xC_16_Ac_3_G_3_) reduced them only slightly.

### Adhesion

The first stage in the formation of biofilms that are difficult to eradicate is the adhesion of microorganisms. Currently, a search is ongoing for surfactants that have the ability to coat various surfaces from which medical devices are made, preventing the adhesion of microorganisms and the formation of biofilms that are difficult to eradicate. For this reason, we investigated the effect of the newly synthesized gemini QAS on the adhesion of *C. albicans* ATCC 10231 to surfaces such as polystyrene, silicone, stainless steel and glass.

**Figure 4:**
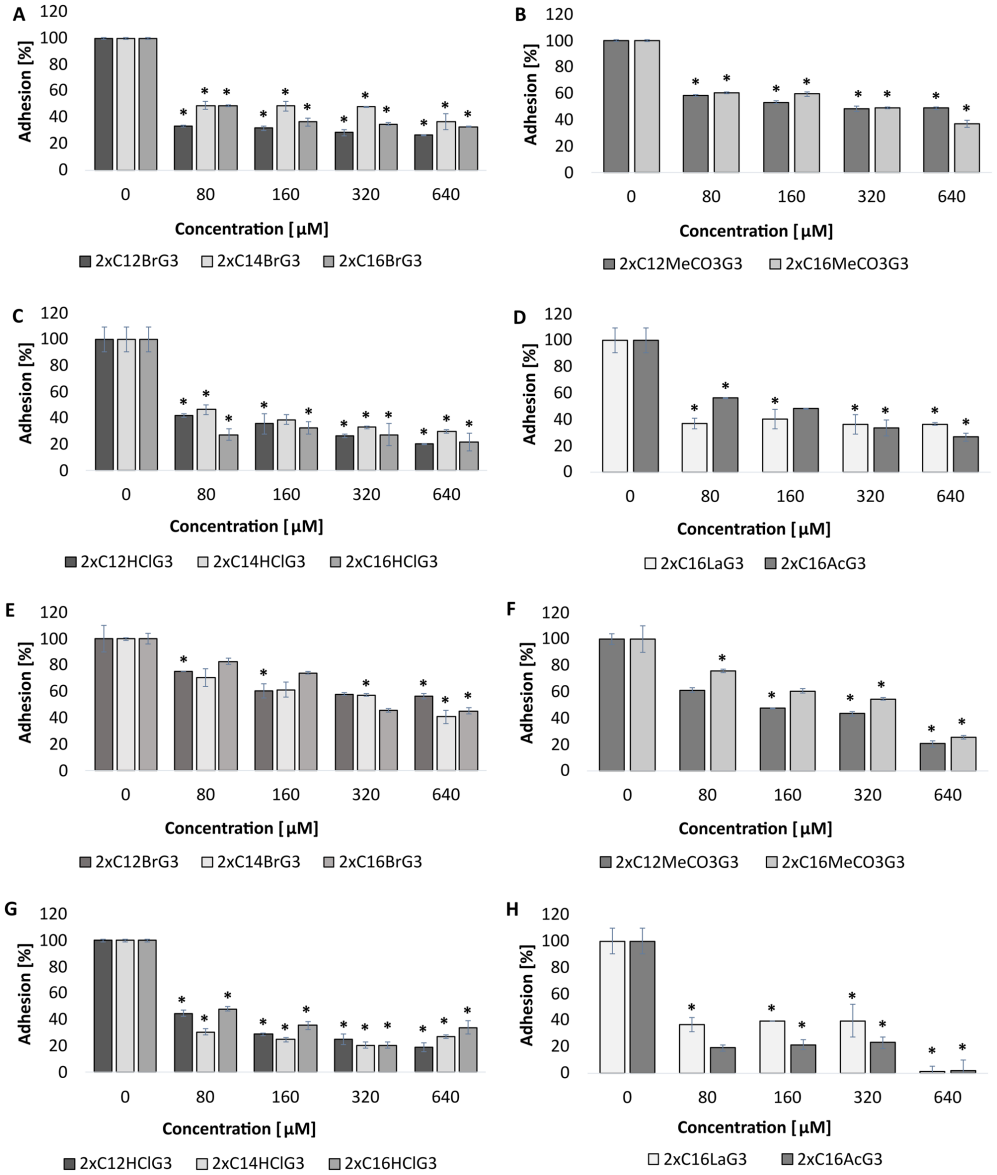
Adhesion of *C. albicans* ATCC 10231 cells to various surfaces: glass (**A**–**D**), silicone (**E**–**F**), after incubation with tested gemini QAS with different alkyl chain lengths, methylene linker (3xCH_2_) and different counterion: ((**A**, **E**—dibromides) 2xC_12_BrG_3_, 2xC_14_BrG_3_, 2xC_16_BrG_3_, (**B**, **F**—dimethylcarbonates) 2xC_12_MeCO_3_G_3_, 2xC_16_MeCO_3_G_3_ (**C**, **G**—dihydrochlorides) 2xC_12_HClG_3_, 2xC_14_HClG_3_, 2xC_16_HClG_3_, (**D**, **H**—dilactate, diacetate) 2xC_16_LaG_3_, 2xC_16_AcG_3_); *significant difference between groups (p < 0.05). Results represent the means ± SD of three independent experiments.

The effect of gemini QAS on the adhesion of *C. albicans* ATCC 10231 cells to the glass surface was determined (Fig. 4A–D). It was found that the tested gemini surfactants reduced this process by approximately 60–80% (p < 0.03). The strongest anti-adhesive activity was demonstrated by dibromides (2xC_12_BrG_3_) (p < 0.004) (Fig. 4A), dihydrochlorides (2xC_12_HClG_3_, 2xC_16_HClG_3_) (p < 0.006) (Fig. 4C) and diacetates (2xC_16_AcG_3_) (p = 0.006) (Fig. 4D), reducing the adhesion of cells of the *C. albicans* strain by approximately 80% at a concentration of 640 µM. The remaining tested compounds also effectively reduced the adhesion of this pathogen to the glass surface (by approximately 60%) (Fig. 4B, D).

The influence of the tested gemini surfactants on the adhesion of *C. albicans* cells to a silicone surface was also examined. It was observed that diacetates (2xC_16_AcG_3_) and dilactates (2xC_16_LaG_3_) at a concentration of 640 µM completely reduced the adhesion of cells of this strain to the tested surface (p = 0.001) (Fig. 4H). However, dihydrochlorides (2xC_12_HClG_3_, 2xC_14_HClG_3_, 2xC_16_HClG_3_) and dimethylcarbonates (2xC_12_MeCO_3_G_3_, 2xC_16_MeCO_3_G_3_) prevented the adhesion of these microorganisms by approximately 80–90% (p < 0.03) (Fig. 4F, G). Dibromides reduced the adhesion of *C. albicans* by approximately 40–60% (p < 0.04) (Fig. 4E).

**Figure 5:**
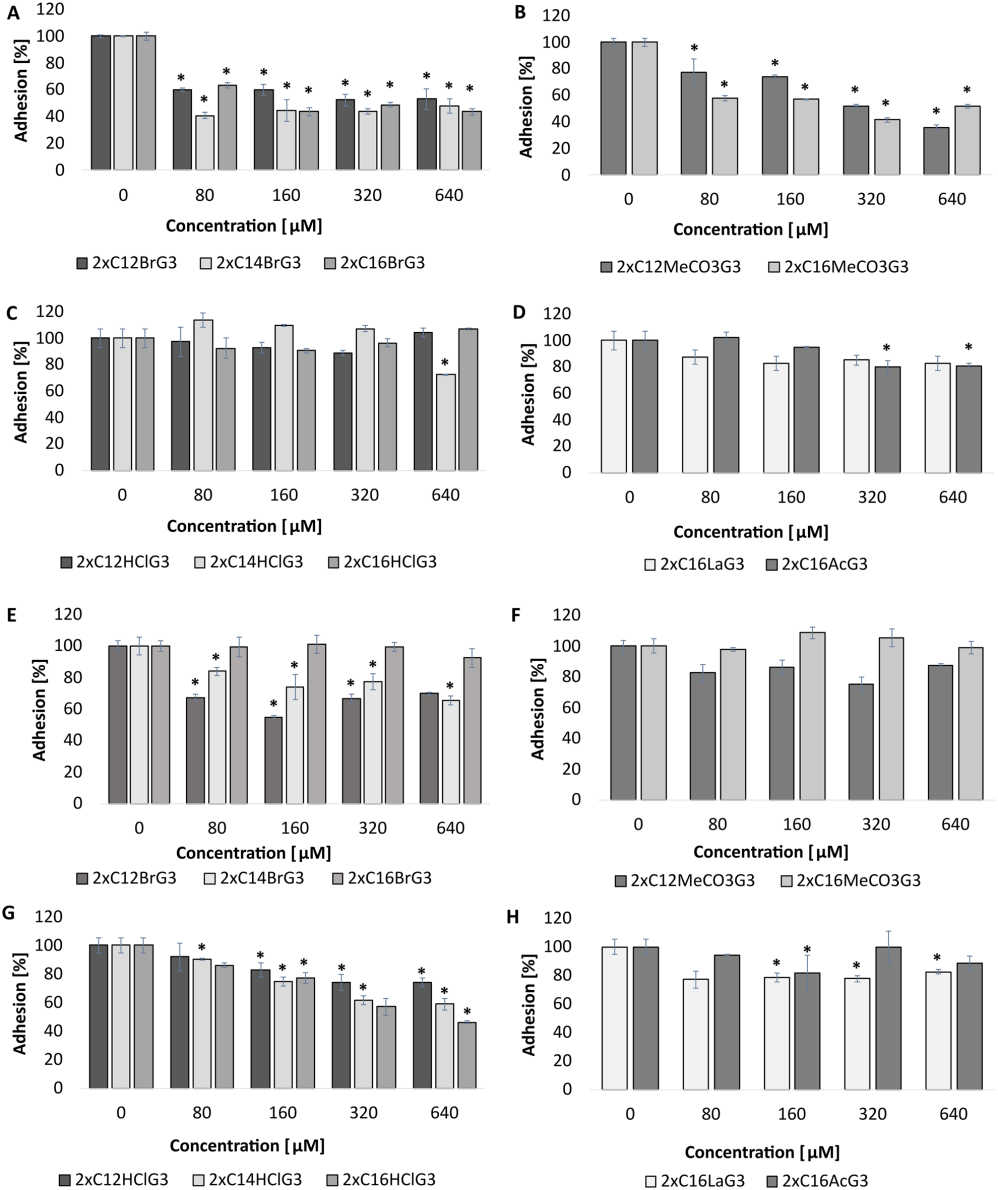
Adhesion of *C. albicans* ATCC 10231 cells to various surfaces: stainless steel (**A**–**D**), polistyrene (**E**–**F**), after incubation with tested gemini QAS with different alkyl chain lengths, methylene linker (3xCH_2_) and different counterion: ((**A**, **E**—dibromides) 2xC_12_BrG_3_, 2xC_14_BrG_3_, 2xC_16_BrG_3_, (**B**, **F**—dimethylcarbonates) 2xC_12_MeCO_3_G_3_, 2xC_16_MeCO_3_G_3_ (**C**, **G**—dihydrochlorides) 2xC_12_HClG_3_, 2xC_14_HClG_3_, 2xC_16_HClG_3_, (**D**, **H**—dilactate, diacetate) 2xC_16_LaG_3_, 2xC_16_AcG_3_); *significant difference between groups (p < 0.05). Results represent the means ± SD of three independent experiments.

The anti-adhesive effect of the tested compounds was also determined on the surface of stainless steel. Dibromide (2xC_16_BrG_3_) and dimethylcarbonate with 12-carbon alkyl chains had the strongest effect on this surface (approximately 60% inhibition of adhesion) (p < 0.04) (Fig. 5A, B). Dibromides with 12- and 14-carbon alkyl chains and dimethylcarbonate (2xC_16_MeCO_3_G_3_) prevented adhesion by approximately 50% (p < 0.01) (Fig. 5A, B). The remaining QAS did not significantly reduce the adhesion of microorganisms to the stainless steel surface (p < 0.04) (Fig. 5C, D).

The last surface tested for the influence of gemini QAS on the adhesion of *C. albicans* was polystyrene. The tested compounds did not have a strong anti-adhesive effect on this surface. Dihydrochlorides (2xC_12_HClG_3_, 2xC_14_HClG_3_, 2xC_16_HClG_3_) reduced the adhesion of the tested strain by approximately 40% (p < 0.04) (Fig. 5G), and the remaining compounds only by approximately 20% (Fig. 5E, F, H).

### Viability of adhering *C. albicans* cells

Adhesion of *C. albicans* ATCC 10231 to various surfaces may be one of the first steps in the production of biofilms that are difficult to eradicate. For this reason, it was important to determine not only the anti-adhesive properties of the tested gemini QAS, but also the impact of these compounds on the viability of *C. albicans* cells that were adhering.

**Figure 6:**
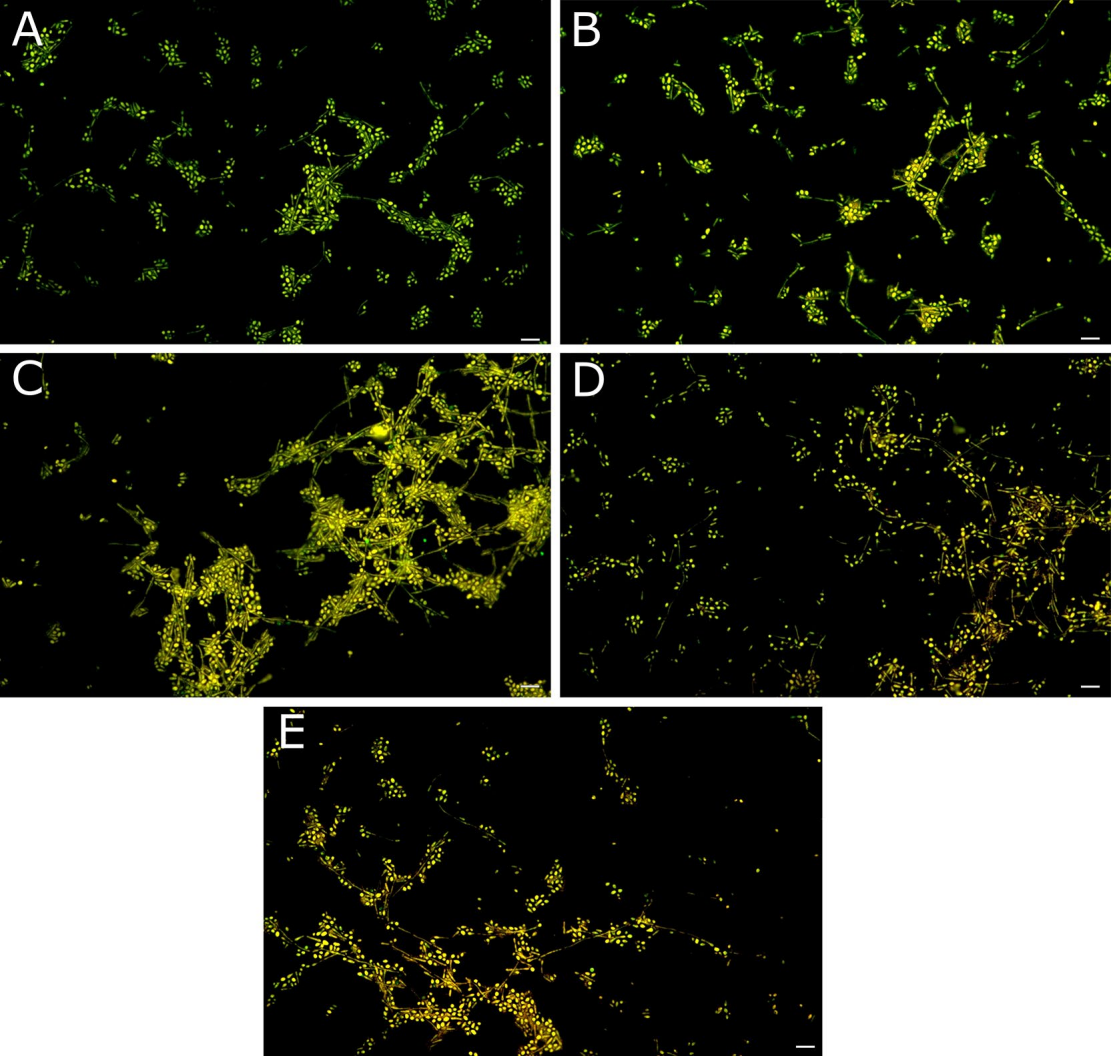
Adhesion viability of *C. albicans* ATCC 10231, on a glass surface, after incubation with compounds with different counterions, alkyl chain lengths and methylene linker (3xCH_2_): (**A**)—negative control (without QAS), (**B**)—2xC_12_BrG_3_, (**C**)—2xC_16_AcG_3_, (**D**)—2xC_12_HClG_3_, (**E**)—2xC_16_HClG_3_. Scale bar = 20 μm.

The effect of the tested surfactants on the survival of adhering *C. albicans* cells was determined using two fluorescent dyes, SYTO 9 and propidium iodide. SYTO 9 is a nucleic acid dye widely used in microbiology that induces green fluorescence in both living and dead microbial cells. Propidium iodide (PI) binds to the DNA of cells with damaged membranes, producing red fluorescence, which reduces the fluorescence caused by the SYTO 9 dye. The experiments demonstrated the effect of the tested gemini surfactants on *C. albicans* cell membranes (Fig. 6A–E). The tested dihydrochlorides (2xC_12_HClG_3_, 2xC_16_HClG_3_) had the strongest effect (Fig. 6D, E).

### Biofilm eradication

Microorganisms are characterized by the ability to produce biofilms—multicellular structures surrounded by an extracellular matrix. Biofilms increase the resistance of pathogens to commonly used disinfectants, making them very difficult to eradicate. QAS have the ability to penetrate the structures of mature biofilm, leading to its destruction.

**Figure 7:**
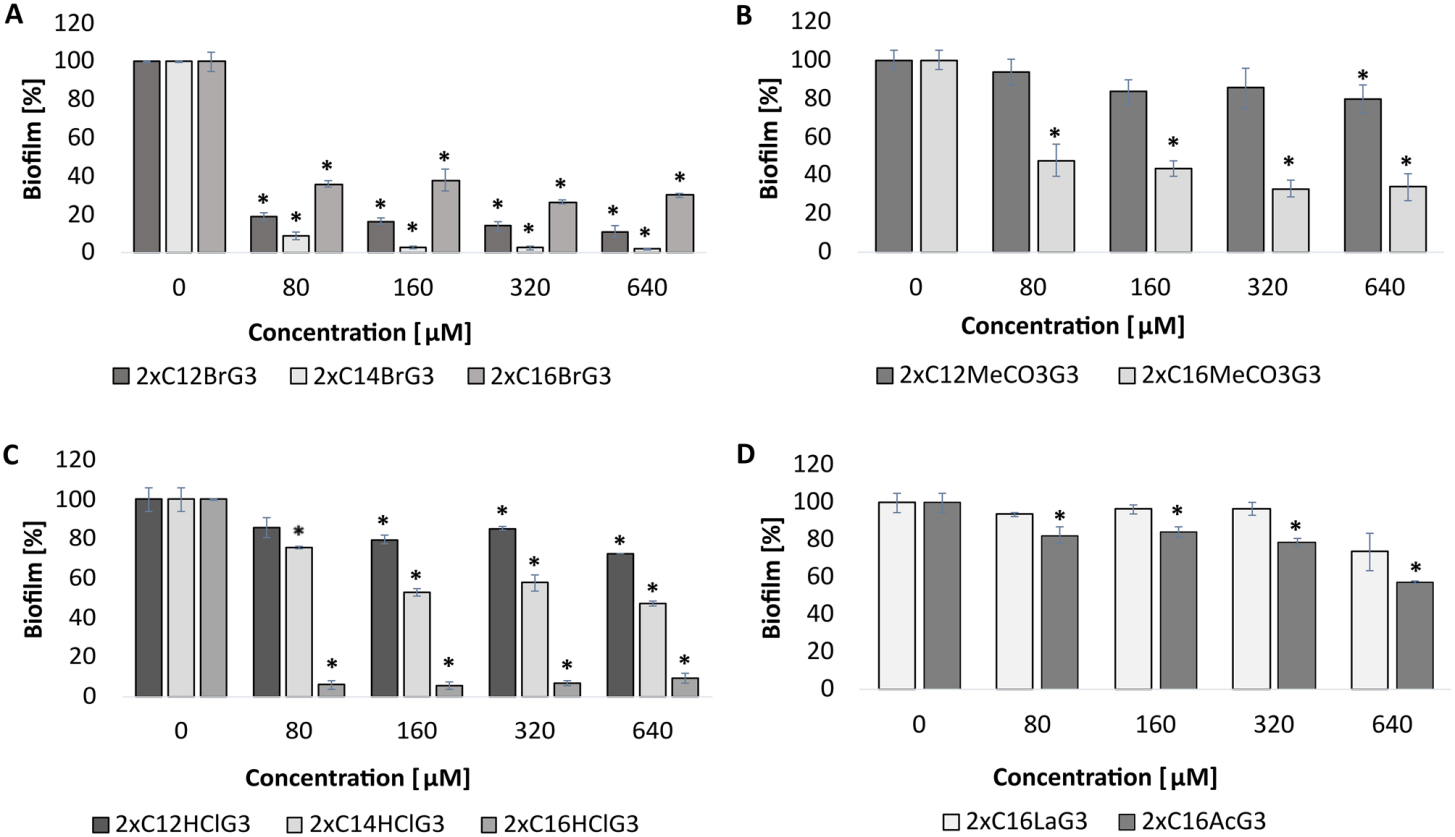
Eradication of *C. albicans* ATCC biofilm after incubation with compounds with different counterions, alkyl chain lengths and methylene linker (3xCH_2_): ((**A**) 2xC_12_BrG_3_, 2xC_14_BrG_3_, 2xC_16_BrG_3_, (**B**) 2xC_12_MeCO_3_G_3_, 2xC_16_MeCO_3_G_3_ (**C**) 2xC_12_HClG_3_, 2xC_14_HClG_3_, 2xC_16_HClG_3_, (**D**) 2xC_16_LaG_3_, 2xC_16_AcG_3_); *significant difference between groups (p < 0.05). Results represent the means ± SD of three independent experiments.

The tested gemini surfactants showed the ability to destroy the biofilm produced by *C. albicans* ATCC 10231 (Fig. 7). Dibromides (2xC_12_BrG_3_, 2xC_14_BrG_3_, 2xC_16_BrG_3_) (p < 0.001) (Fig. 7A), dimethylcarbonates (2xC_16_MeCO_3_G_3_) (p = 0.000) (Fig. 7B) and dihydrochlorides (2xC_12_HClG_3_, 2xC_16_HClG_3_) (p < 0.01) (Fig. 7C) caused approximately 80% destruction of the formed biofilm. However, dihydrochlorides (2xC_14_HClG_3_) (p < 0.005) (Fig. 7C) caused 60% biofilm eradication, and dimethylcarbonates (2xC_12_MeCO_3_G_3_) (p = 0.015) and diacetates (2xC_16_AcG_3_) (p < 0.04) approximately 40% eradication (Fig. 7B, D). The weakest effect was demonstrated by dilactates (2xC_16_LaG_3_), causing biofilm reduction of approximately 20% (Fig. 7D).

### Biofilm viability

Pathogenic microorganisms produce biofilms, which constitute a significant problem among hospitalized patients. These structures are becoming increasingly difficult to eradicate due to their increasing resistance to disinfectants, threatening the lives of many patients around the world. For this reason, it was determined whether the newly synthesized gemini QAS, capable of eradicating biofilms, leads to the death of cells forming bacterial consortia or only reduces their number.

**Figure 8:**
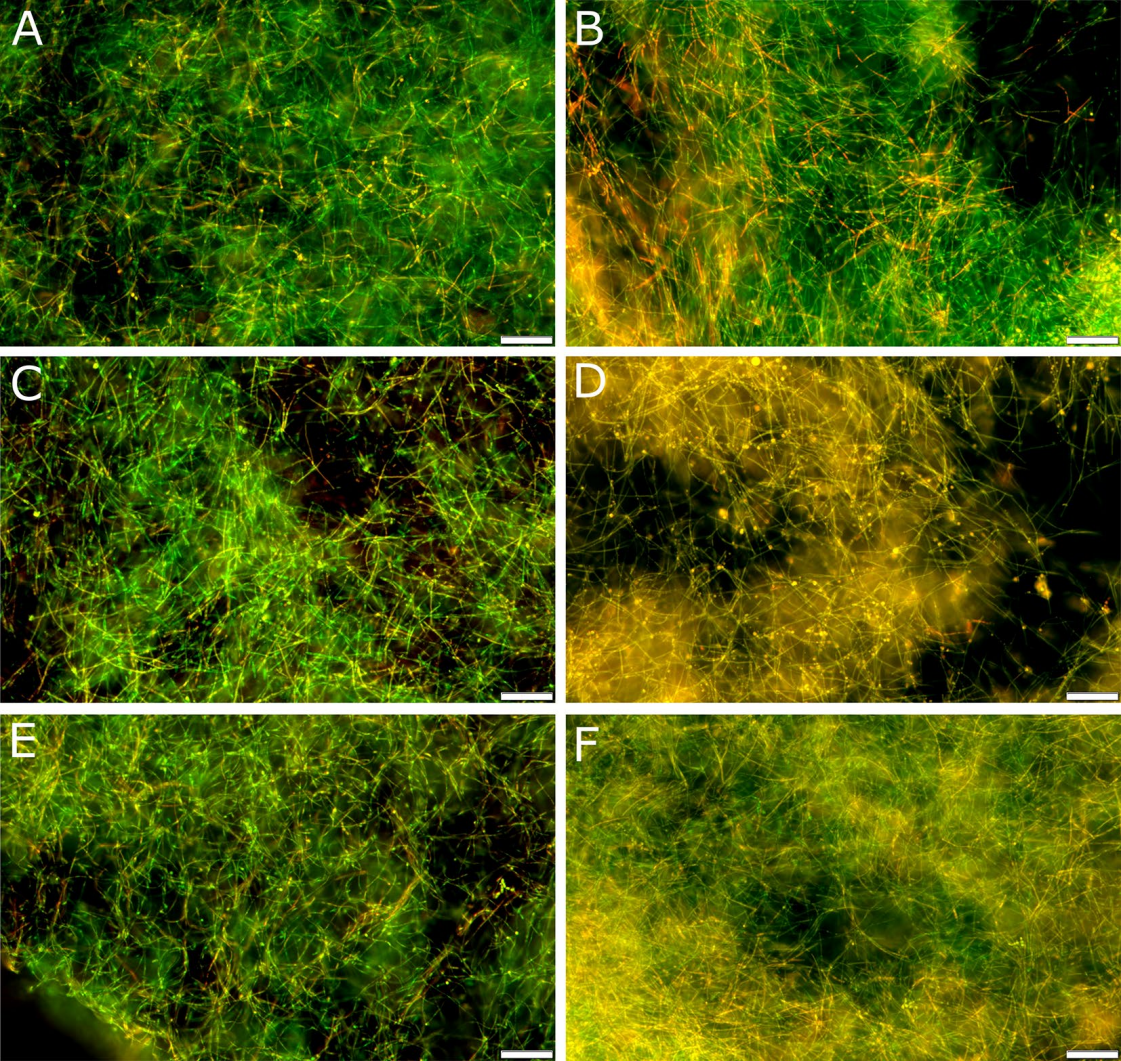
Viability of *C. albicans* ATCC 10231 biofilm, on a glass surface, after incubation with compounds with different counterions, alkyl chain lengths and methylene linker (3xCH_2_): (**A**)—negative control (without QAS), (**B**)—2xC_12_BrG_3_, (**C**)—2xC_14_BrG_3_, (**D**)—2xC_16_BrG_3_, (**E**)—2xC_12_HClG_3_, (**F**)—2xC_16_HClG_3_. Scale bar = 50 μm.

Twenty-four-hour exposure of the biofilm produced by *C. albicans* ATCC 10231 cells to the tested gemini surfactants led to reduction of biofilm compared to control conditions (samples not treated with QAS) (Fig. 8A). The obtained results showed a strong impact of the tested compounds with 16-carbon alkyl chains (2xC_16_BrG_3_ and 2xC_16_HClG_3_) on the survival of the *C. albicans* biofilm*.* As literature data indicate, propidium iodide penetrates into the cells of microorganisms with damaged cell membranes, causing red fluorescence (Fig. 8D, F). The remaining tested compounds did not disrupt the integrity of the cellular structures of *C. albicans* cells present in the biofilm (green fluorescence) (Fig. 8B, C, E).

### Cytotoxicity

In order for the tested gemini QAS to be used in medicine, it was necessary to determine their cytotoxicity. These studies were performed against *S. cerevisiae* Σ1278b ATCC 42800 yeast cells.

**Figure 9:**
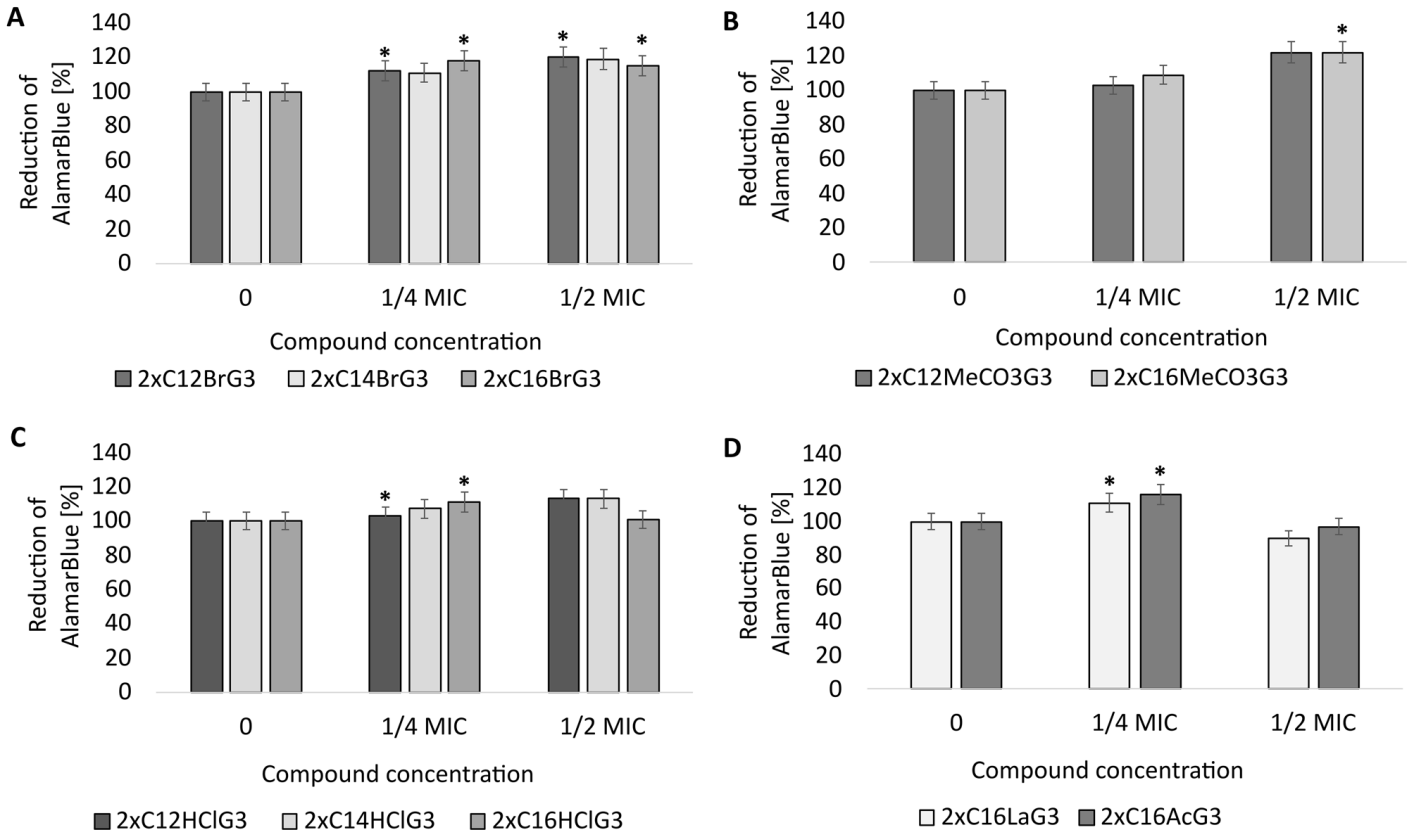
Cytotoxicity of the tested gemini QAS against the yeast *S. cerevisiae* Σ1278b ATCC 42800 after incubation with compounds with different counterions, alkyl chain lengths and methylene linker (3xCH_2_): ((**A**) 2xC_12_BrG_3_, 2xC_14_BrG_3_, 2xC_16_BrG_3_, (**B**) 2xC_12_MeCO_3_G_3_, 2xC_16_MeCO_3_G_3_ (**C**) 2xC_12_HClG_3_, 2xC_14_HClG_3_, 2xC_16_HClG_3_, (**D**) 2xC_16_LaG_3_, 2xC_16_AcG_3_); *significant difference between groups (p < 0.05). Results represent the means ± SD of three independent experiments.

The cytotoxicity of the newly synthesized gemini QAS was determined using resazurin solution (AlamarBlue). The study of the cytotoxic potential of the tested gemini surfactants showed that dibromides (2xC_12_BrG_3_, 2xC_14_BrG_3_, 2xC_16_BrG_3_) (p < 0.05), dimethylcarbonates (2xC_12_MeCO_3_G_3_, 2xC_16_MeCO_3_G_3_) (p = 0.034), dihydrochlorides (2xC_12_HClG_3_, 2xC_14_HClG_3_, 2xC_16_HClG_3_) (p < 0.039), dilactates (2xC_16_LaG_3_) (p < 0.01) and diacetates (2xC_16_AcG_3_) (p < 0.008) at concentrations of ¼ and ½ MIC did not have cytotoxic potential towards the mitochondrial metabolism of *S. cerevisiae* Σ1278b cells (Fig. 9).

### Hemolysis

**Figure 10:**
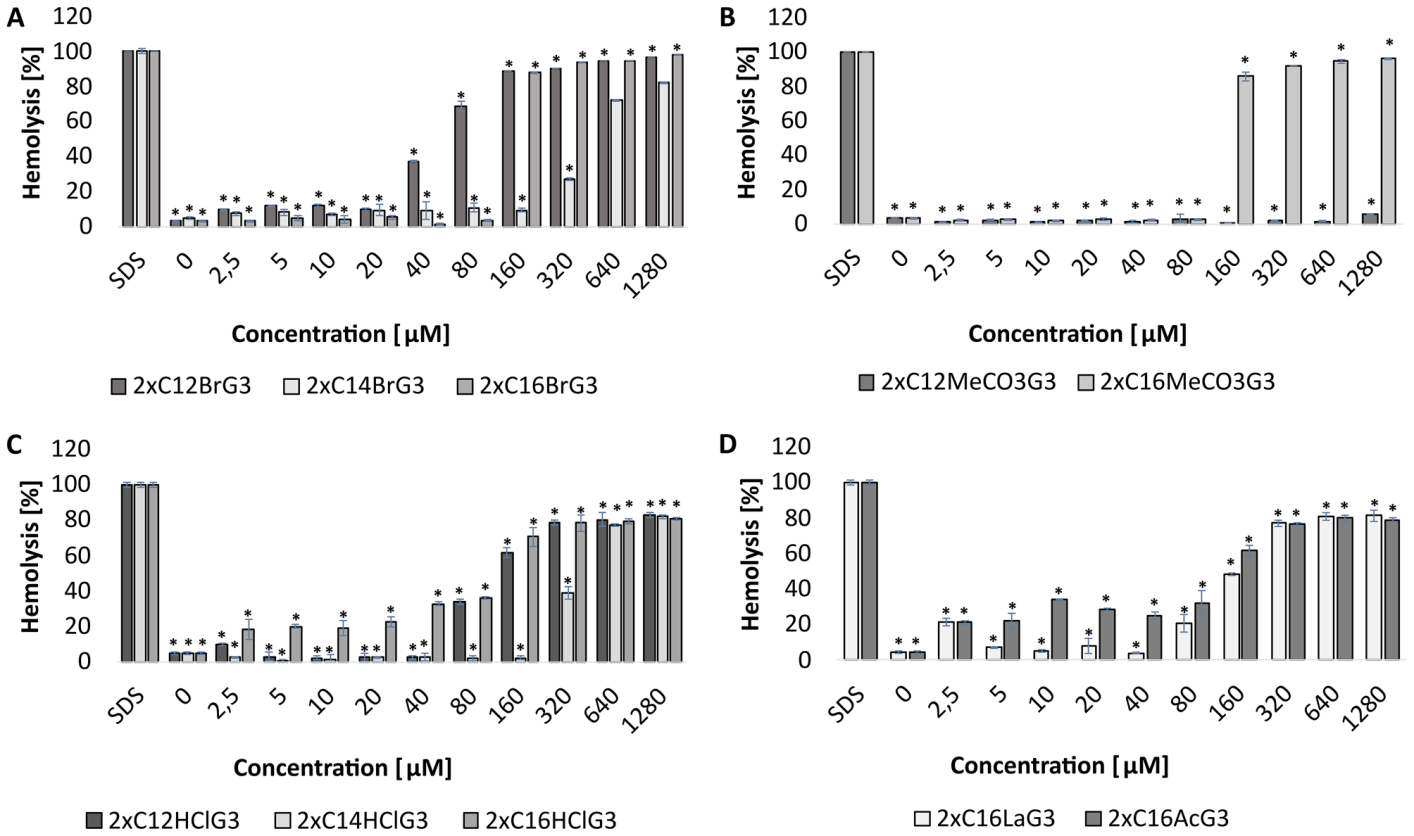
Hemolytic properties of tested gemini QAS with different counterions, alkyl chain lengths and methylene linker (3xCH_2_): ((**A**) 2xC_12_BrG_3_, 2xC_14_BrG_3_, 2xC_16_BrG_3_, (**B**) 2xC_12_MeCO_3_G_3_, 2xC_16_MeCO_3_G_3_ (**C**) 2xC_12_HClG_3_, 2xC_14_HClG_3_, 2xC_16_HClG_3_, (**D**) 2xC_16_LaG_3_, 2xC_16_AcG_3_); *significant difference between groups (p < 0.05). Results represent the means ± SD of three independent experiments.

It was examined whether the tested gemini surfactants cause hemolysis of sheep erythrocytes. The results (Fig. 10) showed that dimethylcarbonate with 12-carbon alkyl chains had no hemolytic effect on sheep erythrocytes (p = 0.000) (Fig. 10B). Dibromide and dihydrochloride with 14-carbon alkyl chains did not cause hemolysis up to a concentration of 160 µM, whereas dibromide (2xC_16_BrG_3_) and dimethylcarbonate (2xC_16_MeCO_3_G_3_) did not cause hemolysis up to a concentration of 80 µM (p < 0.004). Dibromide (2xC_12_BrG_3_) was non-hemolytic up to a concentration of 20 µM and dihydrochloride (2xC_12_HClG_3_) and dilactate (2xC_16_LaG_3_) up to a concentration of 40 µM (p < 0.001). Dihydrochloride (2xC_16_HClG_3_) and diacetate (2xC_16_AcG_3_) up to a concentration of 80 µM caused hemolysis at the level of 20–30%; above this concentration their hemolytic potential increased (p = 0.000).

### Mutagenicity

In order to safely use gemini quaternary ammonium salts in medicine, it was also necessary to determine the mutagenic potential of the tested compounds. Five groups of gemini surfactants with different counterions (dibromides, dihydrochlorides, dimethylcarbonates, diacetates and dilactates) with different numbers of carbon atoms in the alkyl chains and methylene linker (3xCH_2_) were investigated. The tested gemini QAS did not show mutagenic potential against *Salmonella* Typhimurium TA100. Dibromides and dimethylcarbonates (C12) were found to be mutagenic (MR > 2.0), and dibromides (C14, C16) and dimethylcarbonates (C16) were potentially mutagenic (MR > 1.7) at concentrations of 1/2 MIC against *Salmonella* Typhimurium TA98. The remaining tested gemini surfactants did not show any mutagenic potential against this strain (Table 3).

**Table 3:** Mutagenic properties of gemini QAS with different counterion, alkyl chain lengths and methylene linker (3xCH_2_): (2xC12BrG_3_, 2xC14BrG_3_, 2xC16BrG_3_, 2xC12MeCO_3_G_3_, 2xC16MeCO_3_G_3_ 2xC12HClG_3_, 2xC14HClG_3_, 2xC16HClG_3_, 2xC16LaG_3_, 2xC16AcG_3_); ± SD.

Compound	Concentration	*Salmonella* Typhimurium TA98		*Salmonella* Typhimurium TA100	
		Number of colonies	Mutagenicity ratio (MR)	Number of colonies	Mutagenicity ratio (MR)
2xC_12_BrG_3_	½ MIC	174.5 ± 9.19	7.67	308.3 ± 6.87	1.23
	¼ MIC	18.7 ± 2.31	0.82	265.0 ± 7.07	0.95
2xC_14_BrG_3_	½ MIC	48.0 ± 2.83	1.75	197.0 ± 0.71	0.98
	¼ MIC	29.7 ± 2.08	1.08	199.0 ± 1.41	0.99
2xC_16_BrG_3_	½ MIC	42.0 ± 5.57	1.85	418.0 ± 2,83	1.50
	¼ MIC	34.3 ± 5.69	1.51	380.0 ± 1.37	0.00
2xC_12_MeCO_3_G_3_	½ MIC	59.0 ± 2.65	2.59	438.0 ± 2.83	1.57
	¼ MIC	35.8 ± 2.63	1.57	243.0 ± 8.49	0.87
2xC_16_MeCO_3_G_3_	½ MIC	42.0 ± 2.16	1.85	490.5 ± 3.54	1.76
	¼ MIC	35.3 ± 5.03	1.55	398.0 ± 2.83	1.43
2xC_12_HClG_3_	½ MIC	29.7 ± 1.53	1.08	200.3 ± 1.53	1.00
	¼ MIC	26.3 ± 2.08	0.96	203.0 ± 5.66	1.01
2xC_14_HClG_3_	½ MIC	28.5 ± 0.71	1.04	204.5 ± 6.36	1.02
	¼ MIC	27.5 ± 0.71	1.00	202.5 ± 4.95	1.01
2xC_16_HClG_3_	½ MIC	28.7 ± 4.51	1.04	265.0 ± 2.83	1.32
	¼ MIC	23.0 ± 3.61	0.84	200.0 ± 2.00	1.00
2xC_16_LaG_3_	½ MIC	37.0 ± 3.46	1.35	213.0 ± 4.24	1.06
	¼ MIC	30.7 ± 1.15	1.12	197.7 ± 1.53	0.99
2xC_16_AcG_3_	½ MIC	29.3 ± 2.52	1.07	201.5 ± 3.54	1.00
	¼ MIC	29.0 ± 2.65	1.05	200.0 ± 3.00	1.00
Negative control		27.5 ± 0.70		196.5 ± 0.71	
Positive control		65.0 ± 1	2.85	5116.0 ± 5.66	25.5

Negative control—bacterial dilution was added to top agar without tested compounds.

Positive control—acriflavine (100 μg/mL) for strain TA98 or sodium azide (15 μg/mL) for strain TA100.

## Discussion

QAS are cationic surfactants widely used in industry, medicine and agriculture ^36,37^. These compounds have a broad spectrum of activity against pathogenic microorganisms ^38,39^. They are active against both Gram-positive and Gram-negative bacteria, as well as yeasts and yeast-like fungi ^12^. Their action is based, among other things, on the interaction of the positively charged polar group of the surfactant molecule with the negatively charged cell membrane of microorganisms ^40^.

Our surfactants are characterized by excellent aqueous solubility (they are freely water soluble at concentrations exceeding 1% by weight above 0 °C for shorter chains and at least above 15 °C for longer chains—see Krafft points data in Table 1), due to presence of tertiary amide linker as well as strong cationic hydrophilic headgroups. The main advantage of gemini-type surfactants, in comparison to their linear-type analogues, is their better activity at lower concentrations. Indeed, our newly devised gemini surfactants are characterized by around one or two orders of magnitude lower critical micelle concentrations (typically 10^–4^–10^–7^ mol/dm^3^) when compared to linear-type methylcarbonates, bromides and acetates, comprising tertiary amide linker and the same alkyl chain length (dodecyl, tetradecyl or hexadecyl; typically 10^–2^–10^–3^ mol/dm^3^), published in ^41^. Such behavior opens novel possibilities of their unique performance in anti-adhesive, fungicidal and anti-biofilm action.

The spread of QAS in medicine has led to the development of microbial resistance to commonly used disinfectants ^16,42,43^. In order to search for new surfactants effective against resistant pathogenic microorganisms, new gemini QAS were designed and synthesized.

Our research consisted of determining the biological activity of gemini QAS against yeast cells and yeast-like fungi, both in the planktonic form and forming biofilms. Determination of the minimum inhibitory and fungicidal concentration indicated a significant influence of the alkyl chain length and counterion on the antifungal activity of the tested compounds. The strongest action was observed in surfactants with 12-carbon alkyl chains, having hydrogen chloride and bromide as counterions. According to literature data, compounds with shorter chains show the highest activity against planktonic forms ^44^. The tested gemini QAS with 12-carbon alkyl chains showed fungicidal activity at low concentrations (≥ 2.5 µM) against the yeast *Rhodotorula mucilaginosa* and *Saccharomyces cerevisiae*. In turn, against other tested strains, the remaining tested compounds had very weak or no activity. These results are consistent with the research of Pietka-Otlik et al.^45^, which indicated the lack of sensitivity of *Saccharomyces cerevisiae* and *Rhodotorula glutinis* yeast cells to gemini surfactants. The extension of the hydrocarbon chain led to a decrease in antifungal activity. These results are confirmed by literature data showing the dependence of the antimicrobial activity of the compound on the length of the hydrocarbon chain and the counterion ^43,46^. This effect may be due to the complex structure of the yeast cell membrane. Research indicates a relationship between the length of the hydrophobic chain and the ability of QAS to penetrate the cell membranes of microorganisms ^47^. Moreover, as shown by Vereshchagin et al. ^44^, gemini QAS have a stronger antimicrobial effect than their monomeric counterparts.

Gemini QAS have strong antimicrobial activity against fungi and yeasts as well as gram-positive and gram-negative bacteria ^48^. It has been reported that at low concentrations the compounds are more effective against gram-positive bacteria than against gram-negative bacteria ^49,50^. The characteristic structure of gemini surfactants (two hydrophilic heads and two hydrophobic chains connected by a linker) increases the biological and surface activity of these compounds ^12,46^. These surfactants, compared to their monomeric counterparts, have a better ability to reduce surface tension and a stronger antifungal effect ^51^. Literature data indicate that gemini QAS have stronger antifungal properties, form complexes with DNA more effectively and enable more efficient transfection than their monomeric counterparts ^43,52,53^. As shown by the team of Xie et al. ^54^, the presence of two hydrophilic groups in gemini molecules allows compounds to penetrate more effectively through the cell membranes of microorganisms and enter the cell.

One of the virulence factors of *C. albicans* is the production of hyphae and pseudohyphae ^55,56^. These structures facilitate the adhesion of fungal cells to surfaces and the formation of biofilms. Our research has shown that the tested gemini QAS have the ability to prevent the production of filaments by *C. albicans* after just 12 h of exposure to the compounds. Previous research conducted by our team also confirmed the ability of gemini surfactants to inhibit the formation of these structures ^46,57^.

The present study also determined the effect of the tested gemini QAS on reducing the adhesion of *C. albicans* cells to surfaces such as silicone, glass, stainless steel and polystyrene. Our research demonstrated the ability of the tested surfactants to prevent the adhesion of this strain to the surfaces of silicone, glass and stainless steel. The strongest effects were observed in compounds having methylcarbonate, acetate and lactate as counterions on the silicone surface. Similar results have been obtained by other research teams, indicating the ability of QAS to coat materials such as glass, silicone, stainless steel and polystyrene ^43,57–59^. Modified bacterial cellulose containing QAS based on amino acids and fatty acids had long-lasting and strong antimicrobial properties against pathogens causing skin and wound infections. Moreover, this cellulose showed high biocompatibility, so it could be used as a new class of wound dressings ^60^. Silica nanoparticles modified with quaternary ammonium compounds had anti-adhesive properties, inhibiting the growth of *E. coli* by 96.6% and *S. aureus* by 98.5% compared to unmodified nanoparticles ^61^. Moreover, according to researchers, the adhesion of microorganisms to hydrophobic surfaces is weaker due to the weak bond energy between the cell and the surface ^62^. Research by Labena et al. ^63^ and Gozzelinno et al. ^64^ also showed that QAS have the ability to adhere to the metal surface and create a protective coating on it, preventing the formation of bacterial biofilms and corrosion.

Another important goal of our research was to determine the viability of cells adhering to a glass surface previously exposed to the tested gemini QAS. The use of a mixture of fluorescent dyes binding to the nucleic acid of microorganisms (SYTO 9) and propidium iodide enabled the division of cells into live (green fluorescence) and dead (red fluorescence) ^65,66^. Studies have shown a significant impact of these compounds on the viability of adhered *C. albicans* cells. The team of Lee et al. ^67^ conducted research on coating medical materials with QAS and obtained similar results.

Biofilms produced by *C. albicans* are an increasing problem in hospital environments, causing infections of various types of implants. Due to the high resistance of these microorganisms to the antifungal drugs used and the host's immune mechanisms, it is necessary to remove the implants in order to prevent serious health complications and even death of patients ^68^.

The newly synthesized gemini QAS we tested—dibromides (2xC_12_BrG_3_, 2xC_14_BrG_3_, 2xC_16_BrG_3_), dimethylcarbonates (2xC_16_MeCO_3_G_3_) and dihydrochlorides (2xC_12_HClG_3_, 2xC_16_HClG_3_)—almost completely eliminated the biofilm formed by *C. albicans*. The compounds with the strongest effect were dihydrochloride with 16-carbon hydrocarbon chains and dibromide with 12 and 14-carbon alkyl chains, eradicating biofilm by 80% at a concentration of 40 µM. Zhang et al. ^69^, examining quaternary gemini surfactants, also detected a similar relationship between the chain length and the antimicrobial activity of these compounds.

Research by Costa et al. ^70^ showed that hydrophobicity, counterion and chain length are important for antibacterial activity. The longer the hydrocarbon chain is, the stronger is the antibiofilm activity of the tested compounds. As described by Gundolf et al. ^71^ long chains of compounds can reduce the length of bacterial lipopolysaccharides, while increasing the sensitivity of microorganisms to drugs. Previous research also suggested that due to the amphiphilic nature of QAS, these compounds are able to cover the surface, changing its hydrophobicity. This, in turn, may interfere with the adhesion of pathogens and prevent the formation of biofilms or lead to their eradication ^72^. In addition, gemini QAS have a greater ability to penetrate the cell envelopes of microorganisms than their monomeric counterparts ^73^.

Our research evaluating the survival of the biofilm of *C. albicans* cells on a glass surface revealed a significant impact of the length of the alkyl chain on damaging the cell membranes of microorganisms present in the biofilm. It was found that compounds with 16-carbon alkyl chains had much stronger effects than their shorter counterparts. The red fluorescence visible in the confocal microscopic images indicated the penetration of the dye into *C. albicans* cells with damaged cell membranes ^58^. These results confirm the literature data, indicating the strong antibiofilm effect of QAS having 16 carbon atoms in the alkyl chains. This effect may be due to the increased hydrophobicity of long-chain compounds and their stronger interaction with phospholipids of the *C. albicans* cell membrane ^72^.

In our study, we also determined the cytotoxicity of the tested gemini surfactants against *Saccharomyces cerevisiae* yeast cells. The experiment did not show a decrease in the activity of mitochondria in this strain after incubation with gemini compounds. A similar study by Obłąk et al. ^46^ also indicated a lack of cytotoxic potential of gemini QAS, even those with strong fungicidal activity against planktonic forms of this strain.

In order to use gemini QAS as safe disinfectants, it is necessary to determine their toxicity. Our research on the influence of the tested gemini surfactants on the hemolysis of sheep erythrocytes showed the influence of the counterion and the length of the alkyl chains on the activity of the compounds. Surfactants containing methylcarbonate as a counterion had the lowest hemolytic effect. Moreover, with the increase in QAS concentration and the length of alkyl chains, the hemolytic potential of the tested compounds increased, which is confirmed by literature reports ^41,46,74,75^. Gemini surfactants can penetrate the cell membrane and change its permeability, leading to its lysis ^54^. Gemini quaternary ammonium salts with various counterions (dihydrochlorides, diacetates and dilactates) are not mutagenic. In turn, dibromides and dimethylcarbonates have mutagenic potential. These results indicate the influence of the length of the alkyl chain and the counterion on the mutagenic activity of the tested surfactants, which is confirmed by literature data ^41,46,80^.

Our results confirmed the high application potential of the tested surfactants. Thanks to their strong antifungal, antiadhesive and antibiofilm activity, the newly synthesized gemini QAS can be used as disinfectants. In order for these compounds to be used in medicine, it is necessary to identify their mechanism of action.

## Conclusions

Gemini QAS with 12-carbon alkyl chains (dibromides, dihydrochlorides) inhibit the growth of microorganisms and inhibit the formation of filaments by *C. albicans*. As the length of the hydrophobic chains increases, the activity of these compounds decreases. Additionally, gemini surfactants have the ability to reduce the adhesion of *C. albicans* cells to surfaces such as stainless steel, silicone and glass. The tested gemini QAS (2xC_12_BrG_3_, 2xC_14_BrG_3_, 2xC_16_BrG_3_, 2xC_16_HClG_3_) are characterized by the ability to destroy the formed biofilm, too. However, testing the viability of *C. albicans* cells in the process of adhesion to a glass surface using a fluorescence microscope (SYTO9, PI) showed that dihydrochlorides inhibited cell viability. In turn, the visualization of biofilm viability indicated strong destruction of these structures by compounds with 16-carbon alkyl chains (2xC_16_BrG_3_ and 2xC_16_HClG_3_). The tested gemini QAS do not have cytotoxic potential and do not cause hemolysis up to a concentration of approximately 80 µM.

## Methods

### Materials

Alkanoyl chlorides (dodecanoyl, tetradecanoyl and hexadecanoyl) were obtained from Sigma-Aldrich. Anhydrous lactic acid (99%, crystalline) was purchased from Alfal. All solvents were obtained from Avantor Performance Materials or Stanlab and used as received. Methanol (solvent) for counterions exchange reactions was analytical grade (Avantor Performance Materials) and was additionally dried over CaH_2_ prior synthesis. Deuterated solvents were from Sigma-Aldrich. CMC values of surfactants were measured conductometrically in double distilled water at 25 °C.

### Methods

Quaternary ammonium gemini surfactants with amide linking groups were synthesized in the reaction of tetraamine (N1-(3-(3-(dimethylamino)propylamino)propyl)-N3,N3-dimethylpropane-1,3-diamine—obtained as described by Bradshaw et al. ^76^ with appropriate fatty acid derivative, followed by quaternization with excess of bromomethane or anhydrous hydrochloride gas (for dibromides and dihydrochlorides, respectively). Dimethyl{{3-[N-(3-{N-[3-(dimethylammonium)propyl]hexadecylamido} propyl} hexadecylamido} propyl}} ammonium dilactate and dimethyl{{3-[N-(3-{N-[3-(dimethylammonium)propyl] hexadecylamido} propyl} hexadecylamido} propyl}} ammonium diacetate were obtained by ion exchange in anhydrous methanol, starting from appropriate dimethylcarbonates. For ^1^H NMR and ^13^C NMR analysis surfactants samples were dissolved in appropriate deuterated solvents (CDCl_3_ or DMSO-d_6_) at the concentrations between 5 and 10 mg/cm^3^.

### Synthesis of amideamine derivatives for gemini-type quaternary ammonium bromides and methylcarbonates

N^1^-(3-(3-(dimethylamino)propylamino)propyl)-N^3^,N^3^-dimethylpropane-1,3-diamine (13.46 g, 0.0551 mol) was dissolved in 400 cm^3^ tetrahydrofuran:triethylamine (*v*:*v,* 1:1) mixture, after which the appropriate alkanoyl chloride (0.1101 mol: 24.09 g of dodecanoyl chloride, 27.18 g of tetradecanoyl or 30.27 g of hexadecanoyl chloride) was added dropwise during intensive stirring. Then the reaction mixture was stirred for 12 h at room temperature, followed by filtration of triethylamine hydrochloride, the formed by-product. The filtrate then was evaporated and cautiously dried under reduced pressure for at least 4 h to get dimethyl{{3-[N-(3-{N-[3(dimethylamine)propyl]dodecylamido}propyl} dodecylamido}propyl}}amine or dimethyl{{3-[N-(3-{N-[3-(dimethylamine)propyl]hexadecylamido} propyl} hexadecylamido}propyl}}amine, as viscous liquids. Yield: 99.8%.

### Synthesis of gemini-type quaternary alkylamide ammonium dibromides

Appropriate dimethyl{{3-[N-(3-{N-[3-(dimethylamine)propyl]alkilamido}propyl} alkilamido}propyl}}amine (0.0111 mol: 6.76 g dimethyl{{3-[N-(3-{N-[3-(dimethylamine)propyl]dodecylamido} propyl} dodecylamido}propyl}}amine, 7.38 g dimethyl{{3-[N-(3-{N-[3-(dimethylamine)propyl]tetradecylamido} propyl} tetradecylamido} propyl}} amine or 8.00 g of dimethyl{{3-[N-(3-{N-[3-(dimethylamine)propyl] hexadecylamido} propyl} hexadecylamido}propyl}}amine) was dissolved in 400 cm^3^ of dry tetrahydrofurane and kept at − 20 °C in tightly closed glass vessel for at least 3 h. Into the cooled solution bromomethane (0.0555 mol, 5.3 g) was immediately added, followed by tightly closing of reaction vessel. For synthesis of dimethyl{{3-[N-(3-{N-[3-(dimethylammonium)propyl] tetradecylamido} propyl} tetradecylamido} propyl}} ammonium dibromide ethyl acetate was used as a solvent instead of tetrahydrofurane. The reaction was performed at 0–4 °C for 48 h without any stirring. The precipitates were filtered off, washed with dry tetrahydrofurane and dried over anhydrous P_2_O_5_ in vacuo for at least 12 h. The obtained dimethyl {{3-[N-(3-{N-[3-(dimethylammonium)propyl]dodecylamido}propyl} dodecylamido}propyl}}ammonium dibromide, dimethyl{{3-[N-(3-{N-[3-(dimethylammonium) propyl] tetradecylamido}propyl}tetradecylamido}propyl}}ammonium dibromide and dimethyl{{3-[N-(3-{N-[3-(dimethylammonium)propyl]hexadecylamido}propyl}hexadecylamido}propyl}} ammonium dibromide were kept in desiccator over anhydrous P_2_O_5_ for additional 24 h in order to achieve complete dryness of product. Yield: 76.7% (dimethyl{{3-[N-(3-{N-[3-(dimethylammonium)propyl]dodecylamido}propyl}dodecylamido}propyl}}ammonium dibromide), 81.5% (dimethyl{{3-[N-(3-{N-[3-(dimethylammonium)propyl]tetradecylamido}propyl} tetradecylamido}propyl}}ammonium dibromide) and 87.1% (dimethyl{{3-[N-(3-{N-[3-(dimethylammonium)propyl]hexadecylamido}propyl}hexadecylamido}propyl}}ammonium dibromide).

### Synthesis of gemini-type alkylamide amine dihydrochlorides

Appropriate dimethyl{{3-[N-(3-{N-[3-(dimethylamine)propyl]alkilamido}propyl} alkilamido}propyl}}amine (0.0111 mol: 6.76 g dimethyl{{3-[N-(3-{N-[3-(dimethylamine)propyl]dodecylamido}propyl}dodecylamido}propyl}}amine, 7.38 g dimethyl{{3-[N-(3-{N-[3-(dimethylamine)propyl]tetradecylamido}propyl}tetradecylamido}propyl}} amine or 8.00 g of dimethyl{{3-[N-(3-{N-[3-(dimethylamine)propyl]hexadecylamido}propyl} hexadecylamido}propyl}}amine) was dissolved in 150 cm^3^ of chloroform and cooled to around 5 °C. The solution was kept in a reaction vessel equipped with cooling jacket and slowly stripped with freshly generated and dried gaseous hydrochloride in order to obtain near complete dissolution of gas in chloroform. The reaction vessel was than kept at around 0 °C for at least 24 h in order to assume reaction completion. After that a certain amount of cold acetone was introduced until slight turbidity appeared. Mixture was kept at 0–5 °C and the formed precipitate was filtered up. The obtained dimethyl{{3-[N-(3-{N-[3-(dimethylamine)propyl]dodecylamido}propyl}dodecylamido}propyl}} amine dihydrochloride, dimethyl{{3-[N-(3-{N-[3-(dimethylamine)propyl] tetradecylamido}propyl}tetradecylamido}propyl}}amine dihydrochloride and dimethyl{{3-[N-(3-{N-[3-(dimethylamine)propyl]hexadecylamido}propyl}hexadecylamido}propyl}}amine) dihydrochloride were purified by crystallization form chloroform/acetone mixture, kept in desiccator over anhydrous P_2_O_5_ for additional 24 h in order to achieve complete dryness of product. Yield: 66.4% (dimethyl{{3-[N-(3-{N-[3-(dimethylamine)propyl]dodecylamido}propyl}dodecylamido} propyl}}amine dihydrochloride), 73.4% (dimethyl{{3-[N-(3-{N-[3-(dimethylamine)propyl] tetradecylamido}propyl}tetradecylamido}propyl}}amine dihydrochloride) and 79.8% (dimethyl{{3-[N-(3-{N-[3-(dimethylamine)propyl]hexadecylamido}propyl} hexadecylamido}propyl}} amine) dihydrochloride).

### Synthesis of gemini-type quaternary ammonium dimethylcarbonates

Appropriate dimethyl{{3-[N-(3-{N-[3-(dimethylamine)propyl]alkilamido}propyl} alkilamido}propyl}}amine (0.0444 mol: 27.04 g dimethyl{{3-[N-(3-{N-[3-(dimethylamine)propyl]dodecylamido}propyl}dodecylamido}propyl}}amine or 32.00 g of dimethyl {{3-[N-(3-{N-[3-(dimethylamine)propyl]hexadecylamido}propyl}hexadecylamido}propyl}}amine) and 0.887 mol (79.93 g) of dimethyl carbonate were placed in pressure reactor, equipped with mechanical stirrer, heating jacket and temperature probe. Reaction mixture was flushed with dry nitrogen four times, in order to remove any oxygen, and heated to 130 °C. Reaction was continued for 6 h at 130 °C and 750 rpm. After reaction completion the final mixture was collected and evaporated to dryness in vacuo. Pure dimethyl{{3-[N-(3-{N-[3-(dimethylammonium)propyl]dodecylamido} propyl}dodecylamido}propyl}}ammonium dimethylcarbonate and dimethyl{{3-[N-(3-{N-[3-(dimethylammonium)propyl]hexadecylamido}propyl}hexadecylamido}propyl}} ammonium dimethylcarbonate were obtained by recrystallization from ethyl acetate. The desired products were filtered off, washed several times with ethyl acetate and dry, cold acetone and dried over anhydrous P_2_O_5_ in vacuo for at least 24 h. Yield: 71.5% (dimethyl{{3-[N-(3-{N-[3-(dimethylammonium)propyl]dodecylamido}propyl}dodecylamido}propyl}}ammonium dimethylcarbonate); 74.6% (dimethyl{{3-[N-(3-{N-[3-(dimethylammonium)propyl] hexadecylamido}propyl}hexadecylamido}propyl}}ammonium dimethylcarbonate).

### Synthesis of gemini-type quaternary alkylamide ammonium dilactate

Dimethyl{{3-[N-(3-{N-[3-(dimethylammonium)propyl]hexadecylamido}propyl}hexadecylamido} propyl}} ammonium dimethylcarbonate (0.0083 mol, 7.50 g) was dissolved in 15 cm^3^ of anhydrous methanol and gentle warmed up to 30–40 °C under stripping with dry nitrogen. Into the obtained solution anhydrous lactic acid (0.017 mol, 1.53 g) dissolved in 15 cm^3^ of anhydrous ethanol was dropwise added under continuous stirring and stripping with nitrogen. Reaction was continued for 5 h at 30–40 °C after addition of lactic acid solution completion. The final mixture was collected and evaporated to dryness in vacuo. Pure dimethyl{{3-[N-(3-{N-[3-(dimethylammonium)propyl] hexadecylamido}propyl}hexadecylamido}propyl}} ammonium dilactate was obtained by recrystallization from ethyl acetate/diethyl ether mixture. The desired product were filtered off, washed several times with diethyl ether and dried over anhydrous P_2_O_5_ in vacuo for at least 24 h. Yield: 95.0%.

### Synthesis of gemini-type quaternary alkylamide ammonium diacetate

Dimethyl{{3-[N-(3-{N-[3-(dimethylammonium)propyl]hexadecylamido}propyl}hexadecylamido} propyl}} ammonium dimethylcarbonate (0.0083 mol, 7.50 g) was dissolved in 15 cm^3^ of anhydrous methanol and gentle warmed up to 30–40 °C under stripping with dry nitrogen. Into the obtained solution anhydrous acetic acid (0.017 mol, 1 cm^3^) dissolved in 15 cm^3^ of anhydrous ethanol was dropwise added under continuous stirring and stripping with nitrogen. Reaction was continued for 7 h at 30–40 °C after addition of acetic acid solution completion. The solvent was evaporated under reduced pressure and the solids were gentle heated to dryness in vacuo. Pure dimethyl{{3-[N-(3-{N-[3-(dimethylammonium)propyl]hexadecylamido}propyl}hexadecylamido}propyl}}ammonium diacetate was obtained by recrystallization from acetonitrille. The desired product were filtered off, washed several times with acetonitrille, followed by drying over anhydrous P_2_O_5_ in vacuo for at least 24 h. Yield: 60.0%.

### Characterization of gemini quaternary type surfactants

Elemental analysis was conducted utilizing Vario EL cube (Elementar, Germany) calibrated on acetanilide. Mass spectra were gain by electrospray ionization mass spectroscopy (ESI–MS) (micrOTOF-Q instrument; Bruker Daltonics, Germany). The ESI–MS aparatus was operated in the positive ion mode (calibrated with the Tunemix™ mixture; Bruker Daltonics; Germany), followed by spectra analysis by the DataAnalysis 3.4 software (Bruker Daltonics, Germany)—resolution of at least 5 ppm. Proton NMR and ^13^C NMR measurements were performed on a Bruker AMX-500 spectrometer, utilizing CDCl_3_ or DMSO-d_6_ as a solvent. Proton and ^13^C chemical shifts (given in ppm) were calibrated to TMS (δ = 0 ppm) as an internal reference. Krafft points were measured according to procedures described in our previous studies Lamch et al. ^81,82^. Values of CMC for all surfactants were measured utilizing the conductometric method for the compounds’ solutions in milliQ water in the same manner as described in Kula et al. ^41^. For all spectra: ^1^H NMR, ^13^C NMR and ESI–MS as well as appropriate comments see Electronic Supplementary Material.

### Strain and growth conditions

The biological activity of gemini QAS was tested against yeast and yeast-like fungal strains from the ATCC collection and clinical isolates: *Saccharomyces cerevisiae* Σ1278b ATCC 42800, *S. cerevisiae* BY4741 ATCC 201388, *Candida albicans* ATCC 10231, *Rhodotorula mucilaginosa* ATCC 4056 and *C. albicans* 595/20. Yeast and yeast-like fungi were incubated for 18 h in yeast peptone glucose (YPG) medium (1% yeast extract, 1% peptone, 2% glucose) (pH 5.6). The cultures were then centrifuged and suspended in fresh YPG (MIC) or PBS (adhesion, biofilm) medium until the appropriate optical density was obtained.

### Minimum inhibitory concentration and minimum fungicidal concentration

Minimum inhibitory concentration (MIC) and minimum fungicidal concentration (MFC) values were determined based on the modified CLSI M27 protocol^77^. The study was performed using the microdilution method on sterile 96-well titration plates. The *S. cerevisiae* Σ1278b ATCC 42800, *S. cerevisiae* BY4741 ATCC 201388, *C. albicans* ATCC 10231, *C. albicans* 595/20 and *R. mucilaginosa* ATCC 4056 were incubated for 18 h at 28 °C (without shaking), then centrifuged (1000 rcf, 5 min), and the obtained pellet was suspended in fresh YPG medium and optical density (OD) = 0.25–0.3 was determined at a wavelength (λ) of 590 nm. Next, 10 μl of selected compounds at appropriate final concentrations (5–1280 μM) were spotted into the wells of the titration plate. Then, 80 μl of YPG medium and 10 μl of culture with a fixed OD were added. All tests were performed in triplicate. The plate was incubated at 28 °C for 24 h. The YPG medium was a negative control, while the prepared culture not treated with compounds was a positive control. The MIC value was determined using a spectrophotometric reader (BioTek 800 TS Absorbance Reader) at λ = 590 nm. The minimum inhibitory concentration was determined at 90% growth inhibition.

In order to determine the MFC of selected gemini surfactants, 10 μl of culture was transferred to solid YPG medium from the wells of the titration plate in which MIC was observed and from wells with the compound with higher concentrations. The spotted suspensions were incubated at 28 °C for 24 h. After incubation, inhibition of the growth of microorganisms was observed. The fungicidal effect (MFC) of the compound was observed in the absence of fungal cell growth.

### The influence of gemini QAS on the filamentation of *Candida* albicans

The culture of *C. albicans* ATCC 10231 was incubated in YPG medium at 28 °C for 18 h. Then the culture was centrifuged (1000 rcf, 5 min), and the resulting pellet was suspended in fresh YPG medium to obtain a culture with OD = 0.6. Gemini QAS at final concentrations of ½ MIC were added to the resulting culture and incubated at 37 °C with shaking (100 rpm). The formation of filaments was observed using a differential interference contrast microscope (Olympus BX50 DIC Microscope) after 12 and 24 h of exposure to selected surfactants on *C. albicans* cells. The control was a culture not exposed to gemini QAS. The experiment was performed based on a modification of the protocol of Obłąk et al. ^46^.

### Adhesion to polystyrene, stainless steel, silicone and glass

The effect of gemini QAS on the adhesion of *C. albicans* ATCC 10231 was determined using a modification of the method of Obłąk et al. ^58^. The culture of *C. albicans* was incubated with shaking at 37 °C for 18 h. After incubation, the culture was centrifuged (1000 rcf, 5 min), and the resulting pellet was suspended in PBS (pH 7.4) until OD = 0.8 (λ = 590 nm).

1. In the case of testing the polystyrene surface, 100 µl of tested gemini QAS at concentrations of 20–1280 µM was added to the wells of a 96-well titration plate, incubated for 2 h at 37 °C, rinsed with sterile distilled water, and then spotted with 100 µl of the obtained culture and incubated at 37 °C for 4 h. After this time, the culture was removed, the plates were rinsed with sterile distilled water and placed in an oven (60 °C, 20 min). Then, crystal violet (0.1%) was added to the wells of the titration plate, stained for 5 min and rinsed with sterile distilled water until all traces of violet were removed from the rinsed water. Then the washing mixture (1% SDS, 50 mmol HCl, 100% isopropanol) was added. The absorbance of the samples was read using a spectrophotometer (BioTek 800 TS Absorbance Reader) at λ = 590 nm. The positive control was the culture wells to which the tested gemini QAS were not added, and the negative control was the medium. The experiment was performed in 3 repetitions.
2. When the tested surface was stainless steel (round washers Ø 0.4 cm), silicone (0.5 cm long fragments of Foley catheters) or glass (1 cm^2^ coverslips), the tested materials were incubated for 2 h at 37 °C with shaking (100 rpm) in the presence of the tested gemini QAS at concentrations of 80–640 µM. Then, the materials were rinsed with sterile distilled water, transferred to tubes with culture and incubated for 4 h at 37 °C with shaking (100 rpm). After incubation, the materials were rinsed with sterile distilled water. The next part of the experiment was performed using one of two methods:

- Crystal violet (0.1%) was added to the tested samples, stained for 5 min, and rinsed with sterile distilled water until the rest of the violet was removed from the rinsed water. Next, the washing mixture (1% SDS, 50 mmol HCl, 100% isopropanol) was added and the absorbance of the samples was read using a spectrophotometer (BioTek 800 TS Absorbance Reader) at λ = 590 nm. The positive control was the culture wells to which the tested gemini QAS were not added, and the negative control was the medium. The experiment was performed in 3 repetitions.
- The tested materials were transferred to sterile falcons, 5 cm^3^ of PBS was added, then samples were placed on ice and sonicated for 3 min in 3 cycles (Bandelin SonoPuls HD 2070, ~ 30% power). Next, 10 µl of diluted sonicates (10^–3^/10^–4^) were inoculated onto YPG and incubated at 37 °C for 24 h. After this time, the colonies growing on the plates were counted. Positive controls were materials not coated with compounds. The experiment was performed in 3 repetitions. The experiment was performed using a modified version of the Pang et al. method ^78^.

### Viability of adhering *C. albicans* cells

Determination of the effect of the tested gemini QAS on the viability of adhering *C. albicans* cells was performed using Filmtracer LIVE/DEAD Biofilm Viability Kit (Invitrogen).

*C. albicans* ATCC 10231 was cultured in YPG medium at 37 °C for 18 h. After incubation, the culture was centrifuged (1000 rcf, 5 min), and the resulting pellet was suspended in PBS (pH 7.4) until OD = 1.5 (λ = 590 nm).

Next we added 200 µl of selected tested surfactants, in appropriate concentrations, to the wells of the chamber slide plate and incubated it for 2 h. After this time, the compounds were removed and the plate was rinsed with sterile mQ water. Then, 100 µl of the previously prepared *C. albicans* cell suspension was added to each well. The plate was incubated for 4 h at 37 °C. After incubation, the plate was rinsed with PBS, 100 µl of LIVE/DEAD dyes was added, and it was incubated for 30 min in the dark. After this time, the probe was removed and the wells were rinsed twice with PBS. Then, 100 µl of 4% formaldehyde in PBS (pH 7.0) was added and incubated for 30 min in the dark. Formaldehyde was removed and the wells were rinsed twice with PBS. After peeling off the walls of the plate, a drop of 50% glycerol in PBS was placed on each field and a glass slide was placed on it. Adhesion viability of *C. albicans* cells was imaged by confocal microscopy. The negative control was PBS.

### Biofilm eradication

The culture of *C. albicans* ATCC 10231 was incubated with shaking for 18 h at 37 °C, centrifuged (1000 rcf, 5 min), and the pellet was suspended in PBS (pH 7.4) until OD = 0.8. 100 µl of the obtained culture was spotted into the wells of a 96-well plate and it was incubated at 37 °C for 24 h. After incubation, the culture was removed from the wells and rinsed with sterile distilled water. Then, 100 µl of the tested gemini QAS at concentrations of 20–1280 µM were added and incubated for 4 h at 37 °C. After this time, the compounds were removed, and the plates were rinsed with sterile distilled water and placed in an oven (60 °C, 20 min) to strengthen the formed biofilm. Then 100 µl of crystal violet (0.1%) was added and stained for 5 min. The plates were rinsed with sterile distilled water until there were no traces of purple in the rinse water, and 150 µl of washing mixture (1% SDS, 50 mmol HCl, 100% isopropanol) was added. Absorbance was measured at λ = 590 nm (BioTek 800 TS Absorbance Reader). The negative control was the medium, while the positive control was the culture wells to which the tested gemini QAS had not been added. The experiment was carried out based on a modification of the method of Obłąk et al. ^46^. The tested samples were performed in 3 repetitions.

### Biofilm viability

The viability of the biofilm formed by *C. albicans* ATCC 10231 cells was determined using the Filmtracer LIVE/DEAD Biofilm Viability Kit (Invitrogen).

The *C. albicans* culture was incubated in YPG medium at 37 °C for 18 h. After incubation, the culture was centrifuged (1000 rcf, 5 min), and the resulting pellet was suspended in PBS (pH 7.4) to obtain an OD = 1.5 (λ = 590 nm). 100 µl of *C. albicans* cell suspension was placed into the wells of the chamber slide plate and incubated for 24 h at 37 °C. After this time, the culture was removed and the plate was rinsed with sterile mQ water. Then, 200 µl of selected tested surfactants in appropriate concentrations was added to each well and incubated for 4 h. After incubation, the plate was rinsed with PBS, 100 µl of the LIVE/DEAD probe was added, and it was incubated for 30 min in the dark. After this time, the probe was removed and the wells were rinsed twice with PBS. Then, 100 µl of 4% formaldehyde in PBS (pH 7.0) was added and it was incubated for 30 min in the dark. Formaldehyde was removed and the wells were rinsed twice with PBS. After peeling off the walls of the plate, a drop of 50% glycerol in PBS was placed on each field and a glass slide was placed on it. The viability of the biofilm formed by *C. albicans* cells was imaged using a confocal microscope. The negative control was PBS.

### Cytotoxicity

To determine the cytotoxicity of the tested gemini QAS on yeast cells, an experiment was performed using the alamarBlue colorimetric assay to determine the metabolic activity of cells, using the modified alamarBlue Cell Viability Assay Reagent method ^79^. Enzymes of the electron transport system reduce the blue resazurin solution to resorufin, which is fluorescent pink.

The yeast culture *S. cerevisiae* Σ1278b was incubated at 28 °C for 16 h with shaking (100 rpm), centrifuged (1000 rcf, 5 min) and the optical density was obtained, OD = 1.0, λ = 590 nm. On a 96-well titration plate we spotted 160 μl of YPG medium, 20 μl of yeast culture and the tested gemini QAS at final concentrations of ½ and ¼ MIC. The negative control was YPG medium with resazurin solution, and the positive control was resazurin solution with yeast culture. The plates were incubated at 28 °C for 12 h. Then, 20 μl of resazurin solution (alamarBlue) was added to the wells and the plate was incubated for 4 h in the dark. After incubation, the absorbance was measured at λ = 570 nm and λ = 600 nm and the color change of the wells from blue to pink was observed. The experiment was performed in 3 repetitions.

### Hemolysis

The hemolysis test was performed based on the Obłąk et al.^80^ method with modification. Sheep blood (5 cm^3^) was centrifuged (699 rcf, 15 min), washed several times with PBS buffer (pH 7.4), and suspended in PBS. Then, 20 μl of the tested gemini QAS at final concentrations of 2.5–1280 μM and 180 μl of erythrocytes were added to the wells of the titration plate. The positive control was 1% SDS and the negative control was PBS. The plates were incubated for 1.5 h at 37 °C. After incubation, the plates were centrifuged (699 rcf, 15 min) and the supernatant was applied to a new titration plate. Absorbance was measured at λ = 540 nm (BioTek 800 TS Absorbance Reader). The experiment was performed in 4 repetitions.

### Mutagenicity

The mutagenic potential of the tested gemini QAS was determined using the Ames test^83^ using *Salmonella* Typhimurium TA98 and *Salmonella* Typhimurium TA100 with defects in histidine biosynthesis. The culture of *S.* Typhimurium TA98 and *S.* Typhimurium TA100 was incubated with shaking at 37 °C for 18 h, centrifuged (3000 rpm, 5 min) and OD = 1.5 was determined, at a wavelength of λ = 590 nm. The agar was dissolved and cooled to 42 °C. The following were added to the cooled agar: 100 μl of culture, 200 μl of biotin solution (0.031%) with histidine (0.024%) and gemini QAS in final concentrations (½ and ¼ MIC), mixed, poured onto a plate with Davis' minimal medium. The media were incubated for 48 h at 37 °C. The positive control for the *S.* Typhimurium TA98 strain was acriflavin (100 μg/ml), and for the *S.* Typhimurium TA100 strain, sodium azide (15 μg/ml). The negative control was the culture without tested compound and with NaCl (0,9%). After incubation, the colonies were counted and the mutagenicity index (MR) was determined—the ratio of the number of revertants resulting from the mutagenic effect of the tested compound to the number of spontaneous revertants. Experiment was performed in three repetitions.

### Statistical analysis

The results of all the experiments are given as a mean value ± SD (standard deviation) of three independent experiments. The differences in adhesion to different surfaces, biofilm eradication, cytotoxicity and hemolysis of QAS were analyzed with analysis of variance using the Excel MS Office 365 statistical extension. Differences between groups were considered statistically significant for p values < 0.05.

### Supplementary Information

Supplementary Figures.

## Data Availability

The datasets used and/or analyzed during the current study available from the corresponding author on reasonable request. All samples are available from the authors upon request.
