# Supplementary material for: Anti-adhesive, anti-biofilm and fungicidal action of newly synthesized gemini quaternary ammonium salts
Source: Sci Rep. 2024 Jun 19;14:14110. doi: 10.1038/s41598-024-64859-y (PMC11187217; doi:10.1038/s41598-024-64859-y)
Supplement: Supplementary file 1 — Supplementary Figures. [file 41598_2024_64859_MOESM1_ESM.pdf]

ELECTRONIC SUPPLEMENTARY MATERIAL

Anti-adhesive, anti-biofilm and fungicidal action of newly synthesized gemini quaternary ammonium salts

Edyta Mazurkiewicz[1, 3], Łukasz Lamch[2,3], Kazimiera A. Wilk [2] & Ewa Obłąk[1*]

[1] Department of Physico-Chemistry of Microorganisms, Faculty of Biological Sciences, University
of Wrocław, Przybyszewskiego 63/77, 51-148 Wrocław, Poland
[2] Department of Engineering and Technology of Chemical Processes, Faculty of Chemistry, Wrocław
University of Science and Technology, Wybrzeże Wyspiańskiego 27, 50-370 Wrocław, Poland
Corresponding author - [*]Ewa Obłąk: e- mail:ewa.oblak@uwr.edu.pl
[3]These authors contributed equally: Edyta Mazurkiewicz and Łukasz Lamch

1. ^{1}H NMR spectra of the studied compounds

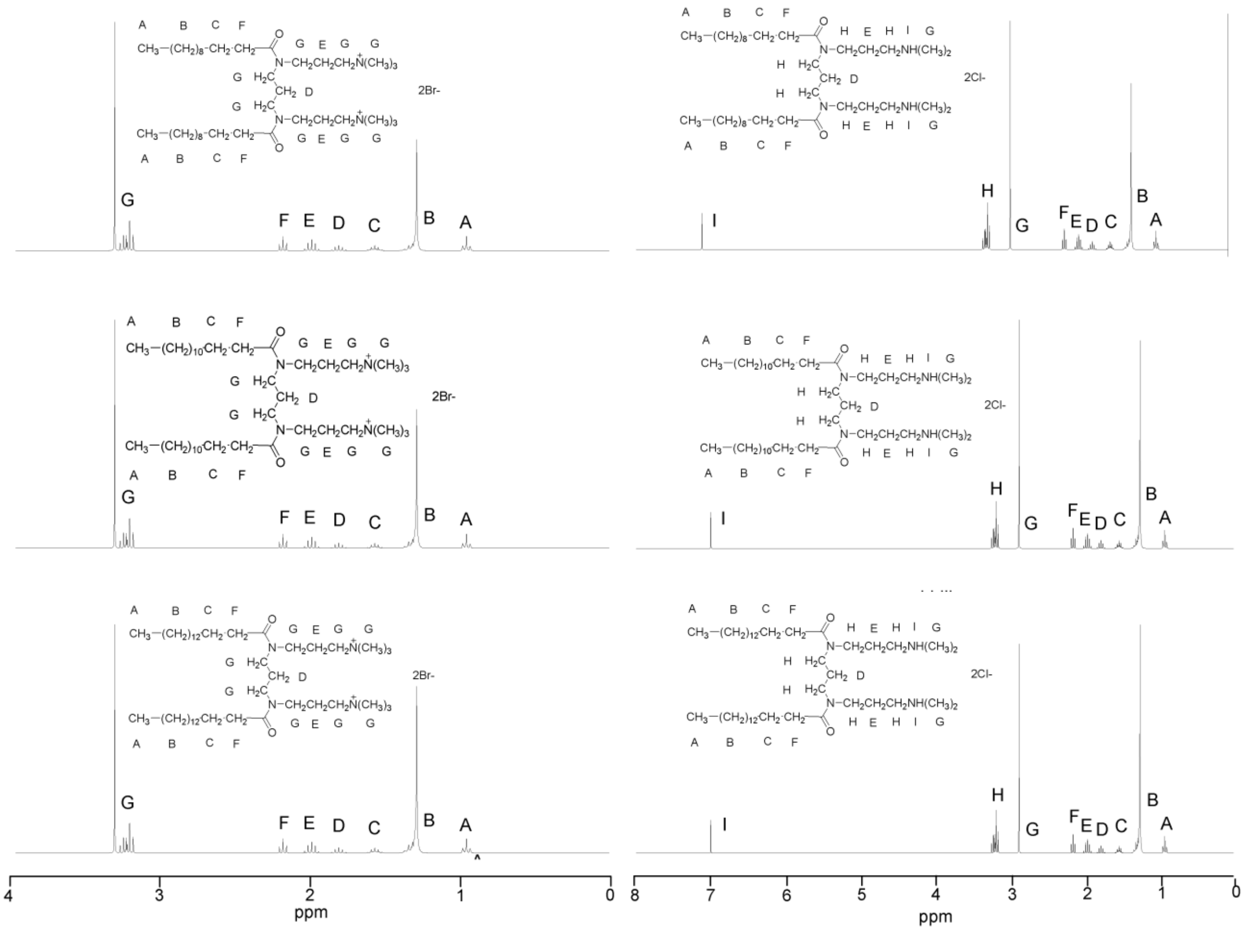

Fig. 1S. ^{1}H NMR spectra of compounds 1 – 6 (see Table 1).

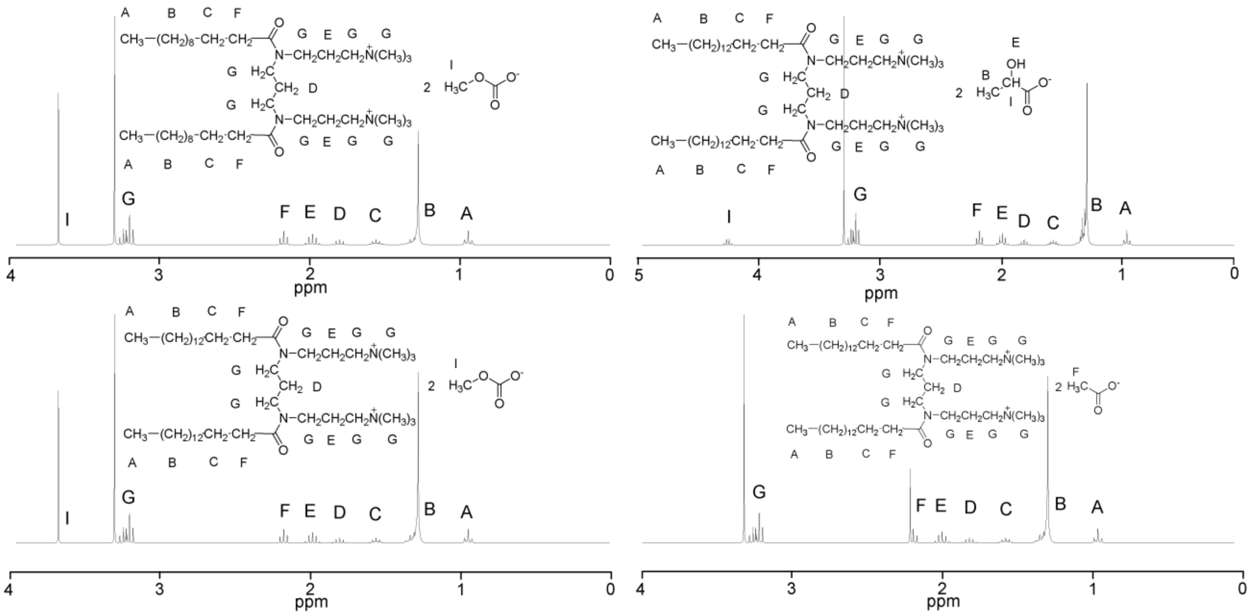

Fig. 2S. ^{1}H NMR spectra of compounds 7 – 10 (see Table 1).

2. ^{13}C NMR spectra of the studied compounds

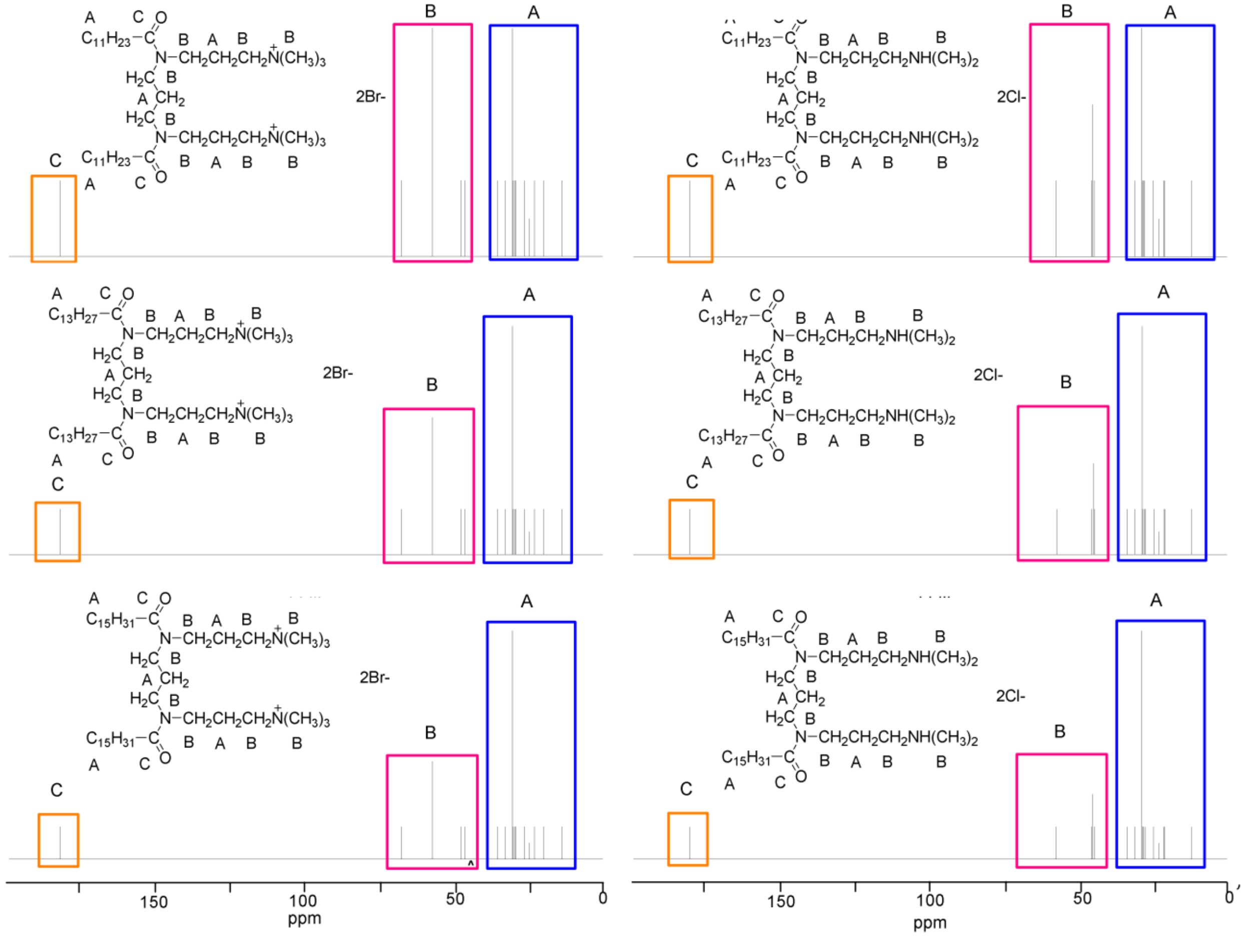

Fig. 3S. ^{13}C NMR spectra of compounds 1 – 6 (see Table 1).

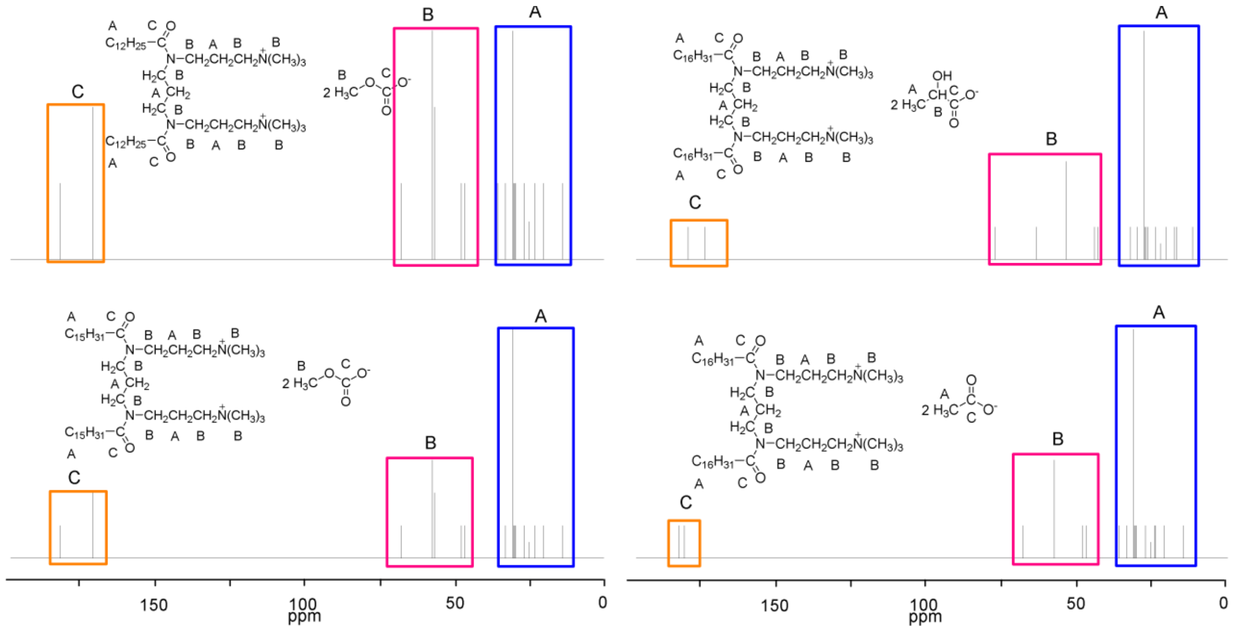

Fig. 4S. ^{13}C NMR spectra of compounds 7 – 10 (see Table 1).

In ^{13}C NMR spectra of all 10 compounds we can see three groups of signals: carbon atoms neighboring only with other carbon atoms (group A, blue rectangle, chemical shifts ca 10 – 35 ppm), carbon atoms neighboring to at least one heteroatom (group B, pink rectangle, chemical shifts ca 45 – 70 ppm) as well as carbonyl carbon atoms (group C, pink rectangle, chemical shifts ca 160 – 180 ppm). These results correspond with ^{1}H NMR, comprising separated methyl group at the end of alkyl chain (chemical shift < 1 ppm), methylene protons in alkyl chain separated from heteroatoms (chemical shifts between 1 and 2 ppm) as well as other protons (chemical shifts > 2 ppm).

2. ESI-MS spectra of the studied compounds

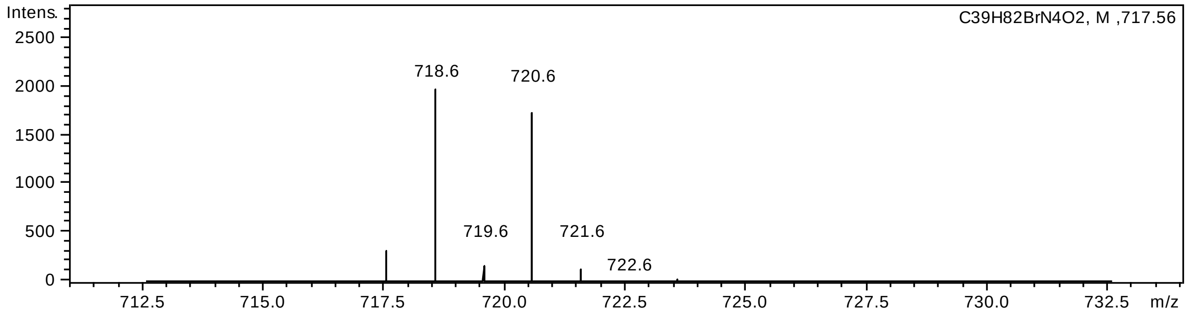

Fig. 5S. ESI-MS spectrum of compound 1 ($2xC_{12}BrG_3$) from Table 1.

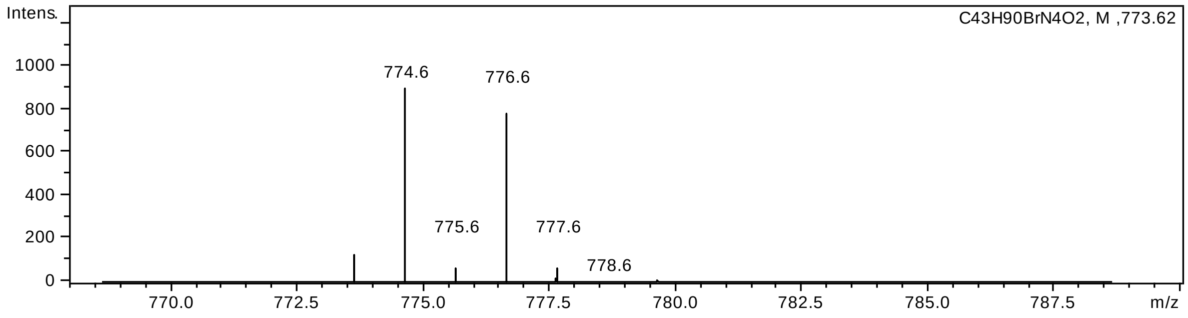

Fig. 6S. ESI-MS spectrum of compound 2 ($2xC_{14}BrG_3$) from Table 1.

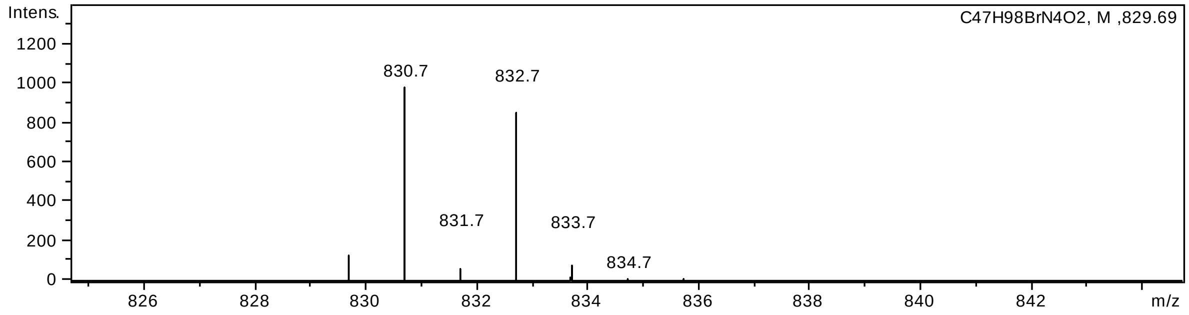

Fig. 7S. ESI-MS spectrum of compound 3 (2x$C_{16}BrG_3$) from Table 1.

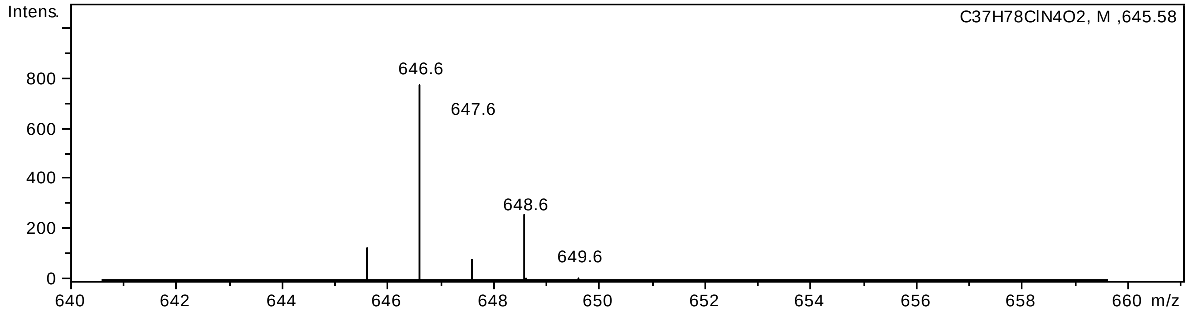

Fig. 8S. ESI-MS spectrum of compound 4 (2x$C_{12}HClG_3$) from Table 1.

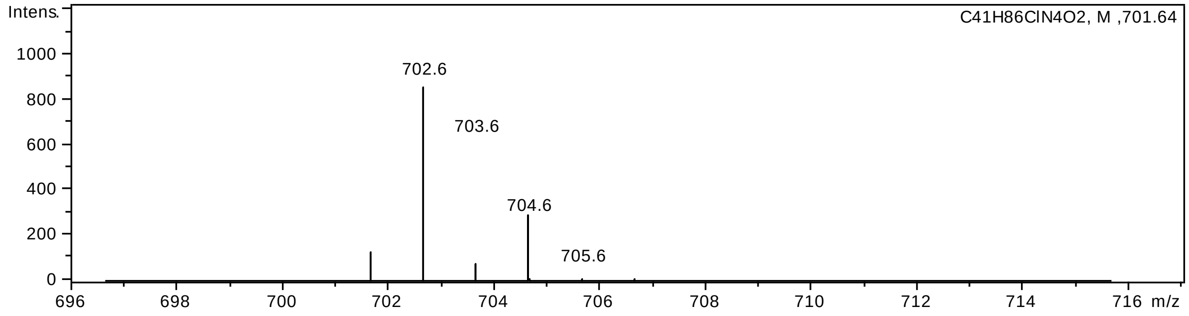

Fig. 9S. ESI-MS spectrum of compound 5 (2x$C_{14}HClG_3$) from Table 1.

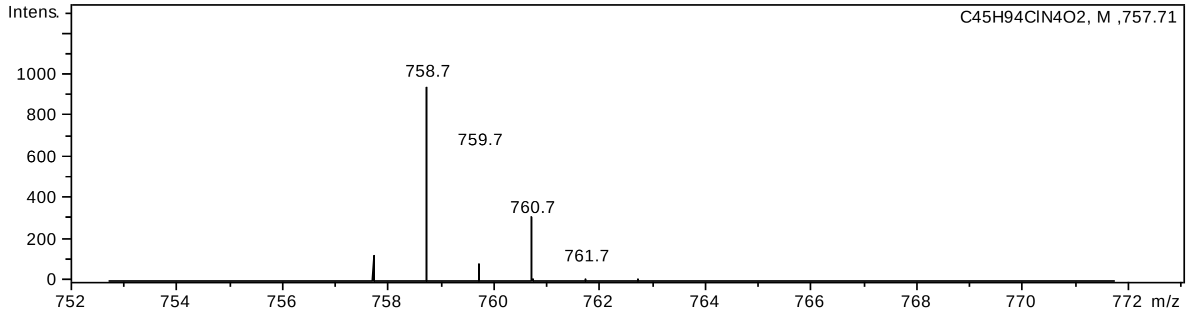

Fig. 10S. ESI-MS spectrum of compound 6 (2x$C_{16}HClG_3$) from Table 1.

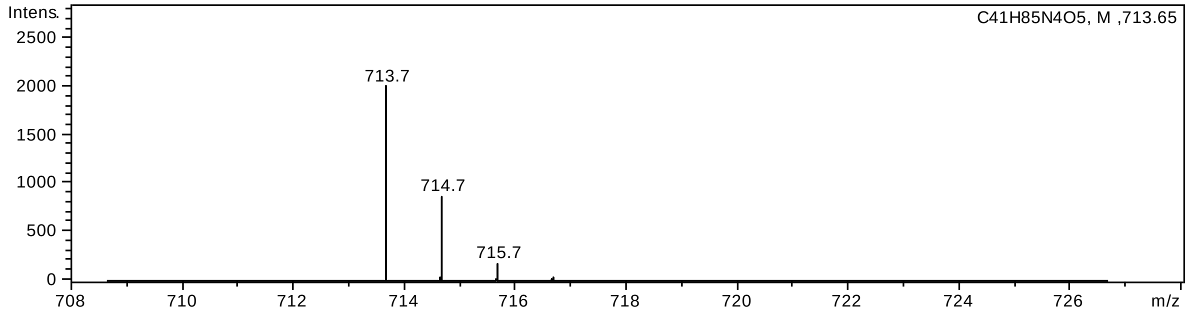

Fig. 11S. ESI-MS spectrum of compound 7 ($2xC_{12}MeCO_3G_3$) from Table 1.

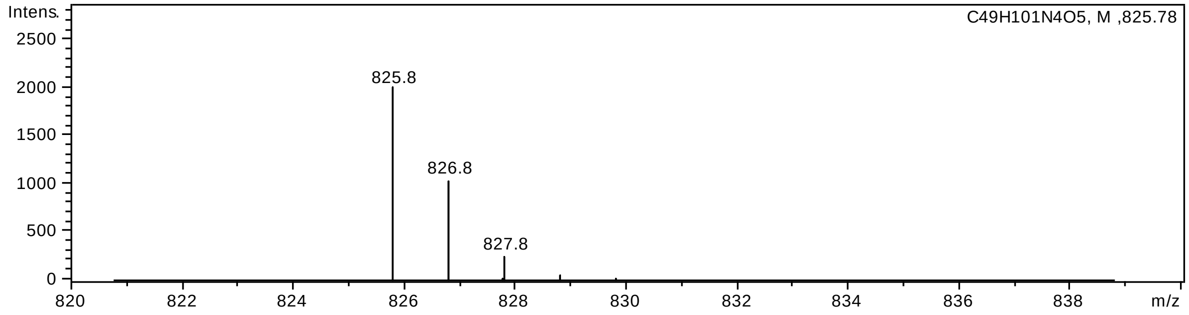

Fig. 12S. ESI-MS spectrum of compound 8 ($2xC_{16}MeCO_3G_3$) from Table 1.

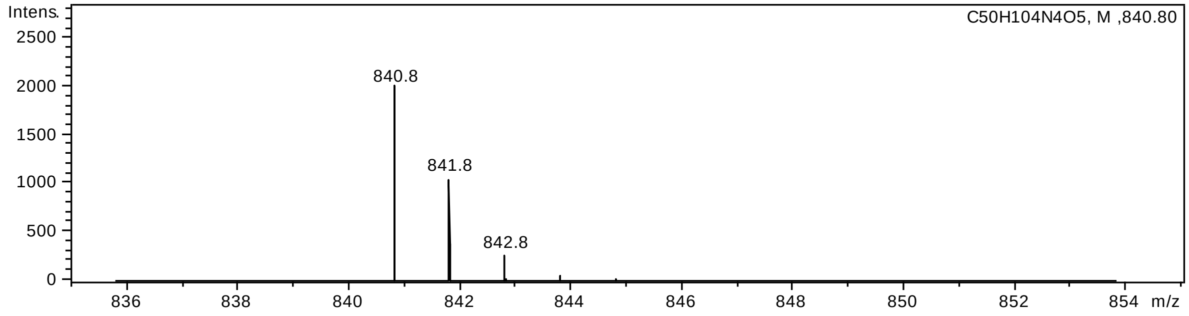

Fig. 13S. ESI-MS spectrum of compound 9 ($2xC_{16}LaG_3$) from Table 1.

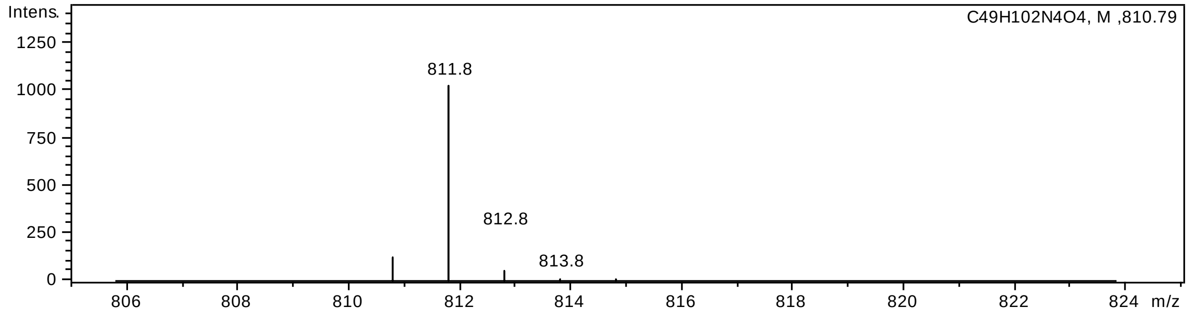

Fig. 14S. ESI-MS spectrum of compound 10 ($2xC_{16}AcG_3$) from Table 1.

ESI-MS spectra of the studied compounds revealed appropriate isotopic pattern characteristic to compounds ionized by detaching of one bromine or chloride atom (of two, present in one particular molecule) as well as compounds comprising exclusively carbon, hydrogen, oxygen and nitrogen atoms (compounds 7 – 10).
